# Life Satisfaction and the Pursuit of Happiness on Twitter

**DOI:** 10.1371/journal.pone.0150881

**Published:** 2016-03-16

**Authors:** Chao Yang, Padmini Srinivasan

**Affiliations:** Computer Science, The University of Iowa, Iowa City, Iowa, United States of America; Beihang University, CHINA

## Abstract

Life satisfaction refers to a somewhat stable cognitive assessment of one’s own life. Life satisfaction is an important component of subjective well being, the scientific term for happiness. The other component is affect: the balance between the presence of positive and negative emotions in daily life. While affect has been studied using social media datasets (particularly from Twitter), life satisfaction has received little to no attention. Here, we examine trends in posts about life satisfaction from a two-year sample of Twitter data. We apply a surveillance methodology to extract expressions of both satisfaction and dissatisfaction with life. A noteworthy result is that consistent with their definitions trends in life satisfaction posts are immune to external events (political, seasonal etc.) unlike affect trends reported by previous researchers. Comparing users we find differences between satisfied and dissatisfied users in several linguistic, psychosocial and other features. For example the latter post more tweets expressing anger, anxiety, depression, sadness and on death. We also study users who change their status over time from satisfied with life to dissatisfied or vice versa. Noteworthy is that the psychosocial tweet features of users who change from satisfied to dissatisfied are quite different from those who stay satisfied over time. Overall, the observations we make are consistent with intuition and consistent with observations in the social science research. This research contributes to the study of the subjective well being of individuals through social media.

## 1 Introduction

The pursuit of happiness is recognized as an unalienable human right in the US constitution. In 1972, Bhutan’s King Jigme Singye Wangchuck rejected GDP as the measure of prosperity and coined gross national happiness (GNH). In 2008 the kingdom introduced a GNH index. Organizations such as the Happy Planet Index [1] monitor global happiness. In academia happiness with its various conceptualizations, determinants, correlates, consequences etc., has long been studied in the social sciences [2]. There is even the journal of Happiness Studies which started in 2000. Somewhat recently happiness research became recognized as important for shedding light on its complement, depression. With all of this collective enthusiasm over happiness it is not surprising to observe a flurry of interest in the study of happiness using social media. However, this is still a nascent field.

Subjective well-being (SWB) is the scientific term for happiness. A concise overview of SWB [3] is provided by Ed Diener a leading researcher in the field. SWB is determined by 3 components: 1) the presence of positive emotions 2) the absence of negative emotions and 3) life satisfaction [4]. The first two determine *affect balance* and are influenced by daily events (eating tasty food, facing traffic congestion etc.). Life satisfaction is a longer-term cognitive assessment of one’s own life. This assessment does not mandate the use of specific criteria such as health, career, family. Instead individuals may use any criteria they think relevant. As examples: ‘*I enjoyed my lunch*’ and ‘*I hate this boring movie*’ reflect positive and negative affect respectively while ‘*I’ve achieved all I wish for in life*’ is about life satisfaction.

Social media research on happiness is mostly limited to studying positive and negative affect [5–13]. In contrast, life satisfaction, our focus, has been studied much less [14–17]. A ‘bridging’ paper [16] tests for correlations between Facebook’s GNH index (measuring positive versus negative words in status updates) and responses to a widely respected life satisfaction survey by Diener et al., [18]. Unsurprisingly they do not find a correlation. This may be because FB status updates relate more to affect while the survey is on life satisfaction. Kross et al. [15] studied Facebook users over a 2-week period and found greater usage leads to lower life satisfaction and lower positive affect. It is unclear though how seriously one should view fluctuations in life satisfaction over a two-week period. Quercia et al. [12, 19] take a different angle. Their case study contextualized in the socioeconomic status of London neighborhoods presents a positive correlation between positivity in tweet sentiment and lower levels of deprivation (measured using IMD, Indices of Multiple Deprivation). Also some LDA based topics correlate positively with deprivation (e.g., celebrity gossip) and others negatively (e.g., vacations). As they state, the connection to subjective well-being research is indirect; prior works link living conditions with subjective well-being and other works link well-being with positivity in user-generated content.

The research of Schwartz et al. [17] studying the language of well-being most directly relates to our work. They analyzed tweets originating in 1,293 US counties for which results from life satisfaction surveys were independently available. Using lexicon features (LIWC and PERMA a special lexicon founded on well-being research [20]) and LDA derived topic features they built models to predict life satisfaction at the county level. Tweet topics and lexical features were of value to the predictors beyond demographic and socio-economic features. Consistent with life satisfaction research in psychology terms and topics such as about physical activity and social engagement related positively to subjective well-being and disengagement words (sleepy, tired) related negatively. A significant difference in our research is methodology. We identify, through passive surveillance, tweeters expressing life satisfaction or dissatisfaction via their tweets. In contrast [17] rely on external survey data to gauge the level of life satisfaction in a county. Individuals surveyed and individuals whose tweets are analyzed may differ. Another difference is their focus on county level aggregations. In contrast we are interested in location independent observations comparing two groups: those whose tweets express life satisfaction and those expressing dissatisfaction. There are significant points of commonality; both explore connections between LIWC and PERMA variables and life satisfaction. We expect and we do achieve mostly consistent results. We present these comparisons in the discussion section.

A challenge when studying SWB using social media (either affect balance or life satisfaction) is to have a reliable method to identify relevant posts. Lexicon approaches are popular, mostly the LabMT lexicon built from Twitter data [5, 9–11] and to some extent ANEW [8], LIWC [12, 17, 19] and also WordNet derivatives [21]. These methods generally use some function of the number of appearances of lexicon words in the post to estimate affect (for example see [9]). Some used OpinionFinder for assessments of emotion as a proxy for ‘happiness’ and found assortivity in Twitter friend networks [6]. Another used the Profile of Mood States (POMS) [7] to examine the impact of events on public mood with tweets. We emphasize: for the most part when social media researchers use the term ‘happiness’ or ‘mood’ they refer to the affect components of SWB. An exception [17] is research with the PERMA lexicon [20]. PERMA designed by Seligman is organized around his model of five elements of well-being: positive emotion, engagement, relationships, meaning in life, and accomplishment. It has 1,524 words arranged in ten categories: a positive and a negative category for each element. While our work is focussed on life satisfaction as reflected in Diener et al., [18] we also consider PERMA in our analysis.

In sum, our goal is to conduct passive surveillance of life satisfaction expressions on Twitter. This is in contrast to direct surveys of life satisfaction via social media as for example done in [16]. For life satisfaction surveillance we need to be able to find posts expressing either satisfaction or dissatisfaction with one’s life. In recent research we presented a methodology to find such posts on Twitter [22]. An overview is in the Methods section. A distinct feature is that our methodology is based on a well respected life satisfaction survey (Diener’s Satisfaction With Life Scale [18, 23]). In essence, we derived a set of template-driven, retrieval strategies from this survey to get the tweets conveying self-ratings of life satisfaction. In our previous paper this strategy achieves recall, precision and F scores between 0.59 and 0.65. This performance is particularly noteworthy given the extremely low signal to noise ratio of life satisfaction expressions in Twitter and the performance of baseline systems. We use this method for finding relevant tweets as the basis of our life satisfaction surveillance strategy. This previous work on methodology [22] was done on 2 days of Twitter data (roughly 4 million tweets/day). Here we present surveillance results from a two-year collection of Twitter data (about 3 billion tweets written in the first person). Our contributions in this paper, which are distinct from the previous work, are as follows.

1. We provide additional validation of our surveillance strategy by directly surveying a sample of Twitter users using the original SWLS survey. Results from the two are consistent (Section 2.2). Also, as expected our two-year trends in life satisfaction posts do not fluctuate with current events (politics, disasters, etc.), (Section 3.1). This differs from reported Twitter trends in affect (e.g., [9]). Crucially, the difference is consistent with the definitions of life satisfaction and affect within subjective-well being [4] and further validates our methods.
2. In static analysis (i.e., disregarding time) we compare users on their Twitter metadata, features related to language use, psychological processes and personal concerns. Users expressing life satisfaction clearly differ from those expressing dissatisfaction (Section 3.2). For example dissatisfied users use more swear and sexual words, and convey more negative emotions such as sadness and anger in their tweets. Our findings are also largely consistent with observations made by Schwartz et al. [17] (Results and Discussion Section).
3. Inspired by the work of Lewinsohn et al. [24] we explore psychosocial variables over time (Section 3.3). Several strong and intuitively acceptable observations arise that are also consistent with findings in Sociology (see also Discussion section). For example, dissatisfied users post more tweets over their timeline on death depression, sadness, anger, anxiety. They also post more negative tweets about home, health and religion.
4. We study users whose tweets indicate a change in position over time: from satisfied to dissatisfied or in the other direction (Section 3.3.2). We observe for example that those who change from satisfied to dissatisfied post more negative tweets (death depression etc.) than the group changing in the other direction. Noteworthy too is that there are even greater differences between satisfied users who change to dissatisfied and satisfied users who stay satisfied with life.

Overall we contribute the first ever study with Twitter expressions of life satisfaction, a key component of subjective well-being. The study complements prior social media research on affect, the other important component of subjective well-being.

## 2 Dataset Description

We used the Twitter Streaming API to collect tweets from Oct. 2012 to Oct. 2014 limited to first person tweets (FP). That is each tweet must have ‘*I*’, ‘*me’*, ‘*my*’ or ‘*mine*’ to increase the likelihood of getting posts conveying self referencing life satisfaction expressions. Contrast the statements: ‘*I hate my life*’ and ‘*You truly have achieved your goals in life*’. This filter differentiates our work from most prior social media research on well being. We collected all metadata such as tweet id, text, utc time, utc offset, geo, place, user id, user name, etc., and user details such as number of followers, number of friends (followings), number of tweets, location eld, etc., (see Table 1).

**Table 1:** Summary of Tweet Dataset.

Time span	2012-10-03 to 2014-09-30
**# Tweets (about 2 years)**	About 3 billion
**# Tweets per day**	About 4 million
**# Unique users per day**	About 3 million
**# Tweets with geo tags per day**	About 100,000
**# Replies per day**	About 600,000
**# Retweets per day**	About 1 million
**# URLs per day (non-unique)**	About 1 million

### 2.1 Validity Checks for Dataset

As per Twitter documentation their Streaming API provides a real time random sample from the complete corpus. We conducted a few validity checks for common sense expectations similar to Dodds et al. [9]. In S1 Fig hourly peak occurrences for tweets with the keywords ‘morning,’ ‘noon’ or ‘evening’ are as expected, e.g., the peak occurrence for ‘morning’ happens around 8 am. Similarly peak occurrences for ‘breakfast,’ ‘lunch,’ and ‘dinner’ are as expected, see S2 Fig. Our dataset has expected properties at least as gauged by these two sets of keywords.

### 2.2 Validity of Surveillance Method

Previously [22], we evaluated our surveillance method by the quality of retrieved tweets using a gold standard set and measures such as precision and recall. We also compared our method with two other popular approaches: using lexicons (labMT and ANEW) and using machine learning. We observed that the lexicon-based methods have low precision. While our machine learning method has better performance than the lexicon approaches, our template-based surveillance method is still much more effective at detecting life satisfaction expressions on Twitter.

Here we present an additional validity test using the SWLS scale as a survey just as intended by its designers. The question we ask is: is there a difference in SWLS life satisfaction scores between satisfied and dissatisfied users identified by our surveillance strategy? Users were selected for survey as follows. Each day we retrieved Class S and D tweets (tweets conveying authors’ life satisfaction and life dissatisfaction respectively) using our strictest retrieval strategy (no intervening words allowed, no words allowed before or after the retrieval template text). See the Methods section for details. Then we randomly selected 120 Class S users and 120 Class D users (users posting the day’s Class S and Class D tweets) as our target users. These numbers were limited by Twitter API constraints. Selected users were invited to take the SWLS survey within 24 hours of their life satisfaction tweet post time. To stimulate participation we promised the chance to win a $50 Amazon gift card. Invitations were sent from 5 different project accounts. The invitation tweet was written in casual language (“win $50 Amazon gift card, complete our survey about life satisfaction”). In the invitation tweet, the user clicked our survey introduction webpage (http://lifesatisfaction.herokuapp.com) which directed the user to the survey webpage at Surveygizmo (http://www.surveygizmo.com) after ensuring that the participant is a valid Twitter user and the one we invited. The estimated time for answering the survey was about 3 minutes. The survey was conducted with IRB approval.

The experiment started Oct. 27th, 2014, and ended on Mar. 16th, 2015. We sent close to 29,890 invitations but received only 137 survey answers (response rate of 0.46%). Due to retweets respondents to our invites included some non-targeted users. After filtering these out 104 respondents remained. This low response rate highlights the extreme difficulty of surveying *specific* Twitter users. Note this is different from conducting a survey to the general Twitter population.

Table 2 summarizes the SWLS scores for Class S and D users. The SWLS gives a score ranging from 5 to 35. Independent t-test on the scores shows that the two classes are significantly different (p value < 0.0001) with the predicted Class S users indicating greater life satisfaction than predicted Class D users.

**Table 2:** Summary of SWLS Survey Results.

	Class S Users identified by our strategy	Class D Users identified by our strategy
# users invited	14,945	14,945
# participants	48	56
Response rate	0.32%	0.37%
SWLS score mean	24.35	19.63
SWLS score std.	6.29	6.67
t test (p value)	**<0.0001**	

### 2.3 Life Satisfaction Tweets and Users Detected

Table 3 summarizes the life satisfaction (LS) tweets and users detected by our surveillance strategy. As expected the proportion of LS tweets is very low. There were about 4 million LS tweets in the 3 billion first person tweet dataset (0.13%) evenly split between the two classes. A Class S (Class D) user is one who posted at least 1 life satisfaction (life dissatisfaction) tweet. At least 17% of users belong to both classes. We analyze such users in a later section. This overlap is at least 17% because the Streaming API used to collect our two-year dataset does not necessarily get the complete set of tweets for a given user. It provides only a random 1% subset of tweets for a query (here the first person query). We overcome this limitation in the next section when we compare user groups.

**Table 3:** Life Satisfaction (LS) Tweets and Users Detected in the Two-Year Dataset. S: Satisfied users, D: Dissatisfied users.

Time Span	2012-10-03 to 2014-09-30
**# Class S tweets**	1,945,198 (0.065%)
**# Class D tweets**	1,908,571 (0.064%)
**# LS tweets per day**	about 4,750
**# unique Class S users**	1,943,832
**# unique Class D users**	1,907,363

## 3 Results

### 3.1 Temporal Trends in Postings of Life Satisfaction Tweets

**Fig 1:**
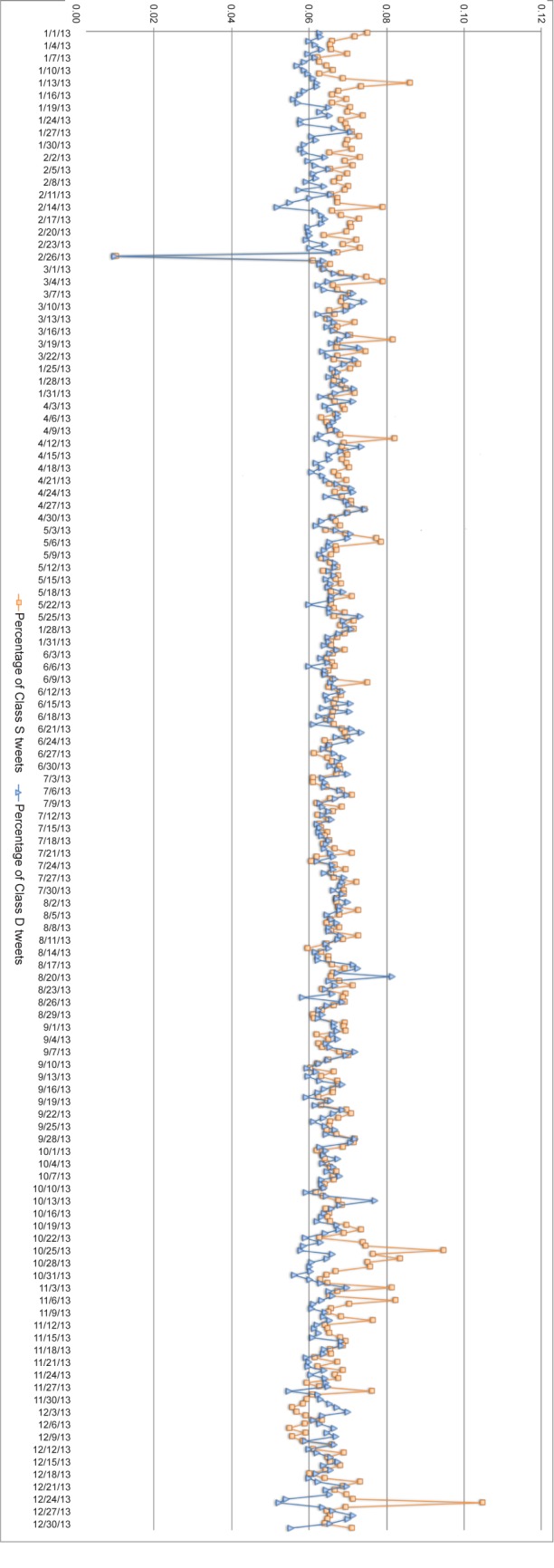
Trend in postings of life satisfaction tweets over one-year period. X axis: week. Y axis: Percentage computed as $\frac{\text{\# Class S (or D) tweets in a day}}{\text{\# First Person Tweets in the day}}\times 100\%$.

Fig 1 shows the distribution of LS tweets for 2013. Time series plots for the full data are in S3 Fig. The figures plot percentage of Class S or Class D tweets over all the FP tweets for each week. Orange (circle) and blue (triangle) lines represent the time series of Class S and Class D tweets respectively.

Overall the key observation is that the two time series show random fluctuations. Political, economic, seasonal and other events do not appear to influence these distributions with very few exceptions. This is noteworthy given the event based fluctuations observed for affect in [9]. We compare these further in the Discussion section. There are significantly more Class S tweets than Class D tweets posted around Christmas and Valentines. We cannot explain the Class D dip on 2013-02-26 or the Class S peek on 2013-10-25. Overall, the time series from 2013-03 to 2013-10 is slightly different from the time series after 2014-04. This may be because the Streaming API unexpectedly returned far more tweets than expected. For example, sometime in March 2014, the API returned 16 million tweets a day—far more than the roughly 4 million till that point. To make the data comparable, we selected 4 million from the 16 million tweets. For every 4 tweets, we selected the first one. Therefore, the time series after March 2014 could be slightly different from the date before. We observed the Streaming API returns 4 million tweets again after Feb. 2015.

### 3.2 Static Differences between Users

We compare users using a static, i.e., a time-independent perspective where the time of post is not considered. Temporal analysis is in the next section. We compare Class S and Class D users in their Twitter features (number of followers, followings and metadata characteristics). We also compare their word usage patterns with Linguistic Inquiry and Word Count (LIWC) software developed by Pennebaker et al. [25] and PERMA lexicon by Seligman [20]. LIWC is popular for calculating the degree to which people use different categories of words and has been applied to a wide array of texts. It has about 80 categories such as ‘*Friends*,’ ‘*Work*,’ and ‘*Money*’ and also sentiment categories such as ‘*Positive/Negative emotion*.’ Some categories are hierarchically arranged as for example ‘*Biological Processes*’ broken down into ‘*body, health, sexual*’ and ‘*ingestion*’. We are interested in categories pertaining to linguistic differences, psychological processes and personal concerns. The PERMA lexicon reflects the view that there are five elements to “well-being”: Positive emotion, Engagement, Relationships, Meaning, Achievement. Each has positive and negative polarity.

Since our dataset does not have the complete set of tweets posted by a user, we generate a new dataset as follows. We randomly selected a subset of Class S and Class D users in our dataset limited to tweets retrieved using our high accuracy strategies (see Methods section). Then we crawled their tweets as many as we can, since Twitter API only returns most recent 3200 tweets for users. In our dataset, we can crawl all the tweets for about 80% users. Then we applied our surveillance method to find all LS tweets posted by these users and made sure that they did not change their LS status from one class to the other. This left us with 14,506 Class S users and 14,743 Class D users with no overlap. We aggregated all crawled tweets for each user into a pseudo document and used these to compare user characteristics. Note because of Twitter API limitations we were able to crawl the full set for only 80% of users. We use Wilcoxon Rank-Sum Test instead of t test, since t test requires “normality” which is not satisfied from our observations. In addition, we use Bonferroni correction given the multiple tests performed.

#### 3.2.1 Followers & metadata differences

Class S and Class D users are similar in numbers of followers and followings. We show their complementary cumulative distribution (CCDF) in S4 and S5 Figs.

Table 4 compares the two classes on metadata. Most differences are significant (Bonferroni-corrected p<0.05). Class S users have longer active lifespan (days between the first and last tweet), use more hashtags but include fewer URLs. Considering a minimum of 5% in difference Class S uses fewer emoticons and get fewer retweets.

**Table 4:** Metadata Features Class S and Class D users differ significantly (Bonferroni-corrected p<0.05) in 8 of 12 features. Significant differences are in italics, differences > 10% are in bold.

Metadata	Class S Avg.	Class D Avg.	Sig.	S—D (%)
*# tweets*	*2,301.0*	*2,401.4*	*.0000*	*-4.3*
***User active days***	***249.1***	***198.0***	***.0000***	***20.5***
Most frq. hour	10.9	11.2	.0135	-2.4
***# urls***	***347.6***	***383.5***	***.0000***	***-10.3***
***# hashtags***	***205.9***	***158.5***	***.0000***	***23.1***
***# unique hashtags***	***107.8***	***84.6***	***.0000***	***21.5***
*# retweets*	*638.2*	*674.5*	*.0000*	*-5.7*
Avg. tweet size	9.9	9.8	.0599	0.3
*# emoticon*s	*1,174.0*	*1,265.8*	*.0000*	*-7.8*
*# pos emoticons*	*159.6*	*160.5*	*.0001*	*-0.5*
# neg emoticons	306.1	292.6	.0026	4.4
# unique words	5,876.3	5,903.1	.9247	-0.5

#### 3.2.2 Language differences

Table 5 compares linguistic style and word usage. Class D users write more often in the present tense, and make more frequent use of personal pronouns and first person singular structures. They also use conjunctions and adverbs more frequently. Class S shows higher use of the exclamation mark. Interestingly, Class D write swear words more frequently. The two groups do not differ in word count (also seen in Table 4, last row) and in their use of first or third person plural structures.

**Table 5:** Linguistic Features Class S and Class D users differ significantly (Bonferroni-corrected p<0.05) in 21 of 25 features. Significant differences are in italics, differences > 10% are in bold.

Categories	Example Words	Class S Avg.	Class D Avg.	Sig.	S—D (%)
Word count		9.5	9.6	.6379	-1.0
*QMark*		*211.8*	*225.5*	*.0000*	*-6.5*
***Exclam.***		***475.3***	***401.8***	***.0000***	***15.5***
*Total function words*		*9,401.1*	*10,099.5*	*.0000*	*-7.4*
*Total pronouns*		*3,539.2*	*3,893.1*	*.0000*	*-9.9*
***Personal pronouns***	***I, them, her***	***2,575.2***	***2,873.8***	***.0000***	***-11.6***
***1st pers singular***	***I, me, mine***	***1,559.5***	***1,790.3***	***.0000***	***-14.8***
*1st pers plural*	*We, us, our*	*113.9*	*115.7*	*.0000*	*-1.6*
*2nd person*	*You, your, thou*	*620.1*	*672.9*	*.0000*	*-8.5*
*3rd pers singular*	*She, her, him*	*181.8*	*195.0*	*.0000*	*-7.3*
3rd pers plural	They, their	99.9	99.8	.0017	0.2
*Impersonal pronouns*	*It, it’s, those*	*964.0*	*1,019.3*	*.0000*	*-5.7*
*Articles*	*A, an, the*	*762.3*	*783.7*	*.0000*	*-2.8*
*Common verbs*	*Walk, went, see*	*3,010.1*	*3,292.5*	*.0000*	*-9.4*
*Auxiliary verbs*	*Am, will, have*	*1,741.3*	*1,895.4*	*.0000*	*-8.9*
*Past tense*	*Went, ran, had*	*440.7*	*466.8*	*.0000*	*-5.9*
***Present tense***	***Is, does, hear***	***2,159.7***	***2,392.9***	***.0000***	***-10.8***
*Future tense*	*Will, gonna*	*187.6*	*203.3*	*.0000*	*-8.4*
***Adverbs***	***Very, really***	***940.2***	***1,047.6***	***.0000***	***-11.4***
Prepositions	To, with, above	1,686.7	1,719.0	.0031	-1.9
***Conjunctions***	***And, but***	***838.6***	***957.2***	***.0000***	***-14.1***
*Negations*	*No, not, never*	*461.4*	*476.3*	*.0000*	*-3.2*
Quantifiers	Few, many	413.8	425.3	.0010	-2.8
*Numbers*	*Second*	*110.1*	*118.6*	*.0000*	*-7.7*
***Swear words***	***Damn, piss***	***220.4***	***246.6***	***.0000***	***-11.9***

#### 3.2.3 Differences in psychological processes

LIWC has psychological process categories such as relating to friends and family, cognitive processes such as certainty, and biological processes such as health and sexuality. Table 6 shows significant differences in most of these categories. Considering only differences above 10% we see that Class D expresses more negative emotion, anger and sadness and also discrepancy (cognitive process). Discrepancy includes words like ‘*should*,’ ‘*would*,’ ‘*expect*,’ ‘*hope*’ and ‘*need*’ that may express determination and aspirations for the future [26, 27]. Also Class D mention significantly more sexual words. Overall, categories under Affective Processes exhibit the largest differences.

**Table 6:** LIWC Psychological Processes. Class S and Class D users differ significantly (Bonferroni-corrected p<0.05) in 24 of 32 features. Significant differences are in italics, differences > 10% are in bold.

Categories	Example Words	Class S Avg.	Class D Avg.	Sig.	S—D (%)
*Social processes*	*Mate, talk, they, child*	*2,058.9*	*2,175.3*	*.0000*	*-5.7*
Family	Daughter, husband	92.7	88.5	.0007	4.6
*Friends*	*Buddy, friend, neighbor*	*52.9*	*55.7*	*.0000*	*-5.4*
Humans	Adult, baby, boy	243.2	247.2	.0006	-1.6
*Affective proc.*	*Happy, cried, abandon*	*1,801.3*	*1,932.4*	*.0000*	*-7.3*
*Positive emotion*	*Love, nice, sweet*	*1,165.7*	*1,220.8*	*.0000*	*-4.7*
***Negative emo.***	***Hurt, ugly, nasty***	***626.5***	***701.2***	***.0000***	***-11.9***
*Anxiety*	*Worried, fearful*	*65.0*	*68.5*	*.0000*	*-5.3*
***Anger***	***Hate, kill, annoyed***	***323.1***	***364.9***	***.0000***	***-12.9***
***Sadness***	***Crying, grief, sad***	***102.1***	***117.0***	***.0000***	***-14.7***
*Cognitive proc.*	*cause, know, ought*	*2,525.9*	*2,700.7*	*.0000*	*-6.9*
*Insight*	*think, know, consider*	*323.1*	*341.0*	*.0000*	*-5.5*
*Causation*	*because, effect, hence*	*259.8*	*282.5*	*.0000*	*-8.7*
***Discrepancy***	***should, would, could***	***350.6***	***395.2***	***.0000***	***-12.7***
*Tentative*	*maybe, perhaps, guess*	*394.0*	*424.5*	*.0000*	*-7.7*
Certainty	always, never	313.8	314.6	.5448	-0.3
Inhibition	block, constrain, stop	99.3	101.3	.0032	-2.0
*Inclusive*	*And, with, include*	*525.6*	*561.6*	*.0000*	*-6.8*
*Exclusive*	*But, without, exclude*	*473.9*	*517.8*	*.0000*	*-9.3*
*Perceptual proc.*	*Observing, heard*	*481.7*	*508.1*	*.0000*	*-5.5*
*See*	*View, saw, seen*	*190.4*	*203.6*	*.0000*	*-6.9*
*Hear*	*Listen, hearing*	*127.5*	*131.4*	*.0000*	*-3.1*
*Feel*	*Feels, touch*	*133.3*	*143.1*	*.0000*	*-7.4*
*Biological proc.*	*Eat, blood, pain*	*667.5*	*731.6*	*.0000*	*-9.6*
*Body*	*Cheek, hands, spit*	*255.5*	*271.5*	*.0000*	*-6.3*
*Health*	*Clinic, flu, pill*	*118.3*	*124.8*	*.0000*	*-5.5*
***Sexual***	***Horny, love, incest***	***242.6***	***285.4***	***.0000***	***-17.6***
Ingestion	Dish, eat, pizza	104.0	105.0	.0119	-1.0
Relativity	Area, bend, exit, stop	2,344.1	2,388.7	.0751	-1.9
*Motion*	*Arrive, car, go*	*368.3*	*398.0*	*.0000*	*-8.1*
Space	Down, in, thin	873.1	869.4	.2821	0.4
Time	End, until, season	1,044.7	1,064.8	.0203	-1.9

**Fig 2:**
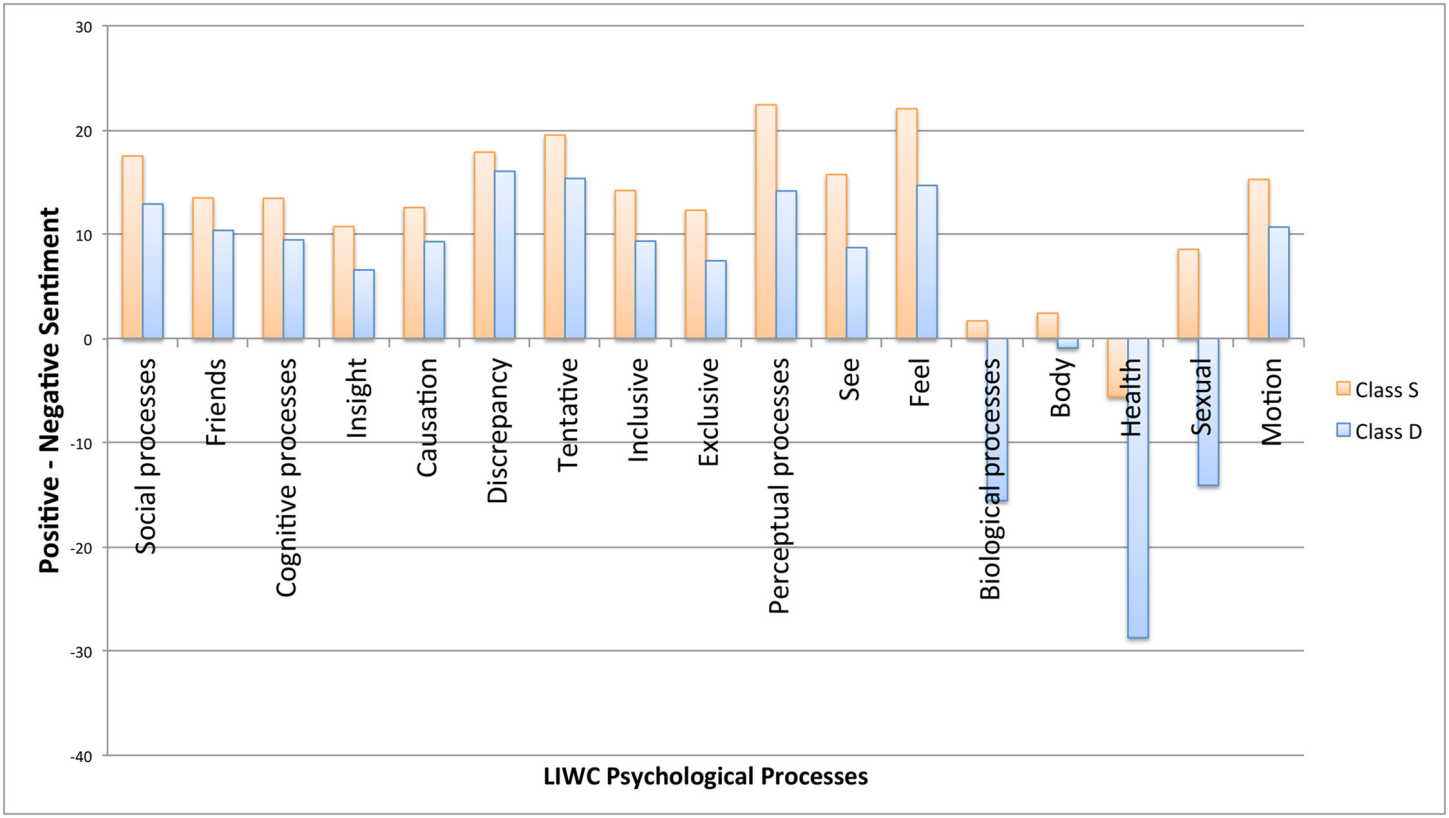
Comparison of user classes on sentiment and LIWC psychological processes. X axis: Psychological process categories. Y axis: % of positive sentiment tweets − % of negative sentiment tweets. Tweets from Class S users are more positive than negative compared to tweets from Class D users in all categories. The biggest differences are in biological processes, sexuality and health.

A limitation is that while we see significant differences in the extent to which categories such as Family, Friends and Health are discussed, we do not know whether negative or positive emotion dominates. Thus we overlay sentiment analysis and consider the relative distribution of sentiment in Fig 2. We do this for each category with a minimum difference of 5% that does not have a natural sentiment loading (e.g., anxiety). Interestingly, in each category Class S reflects more positive sentiment. This is particularly pronounced for Health, Sexual and Biological Processes.

#### 3.2.4 Differences in personal concerns

**Fig 3:**
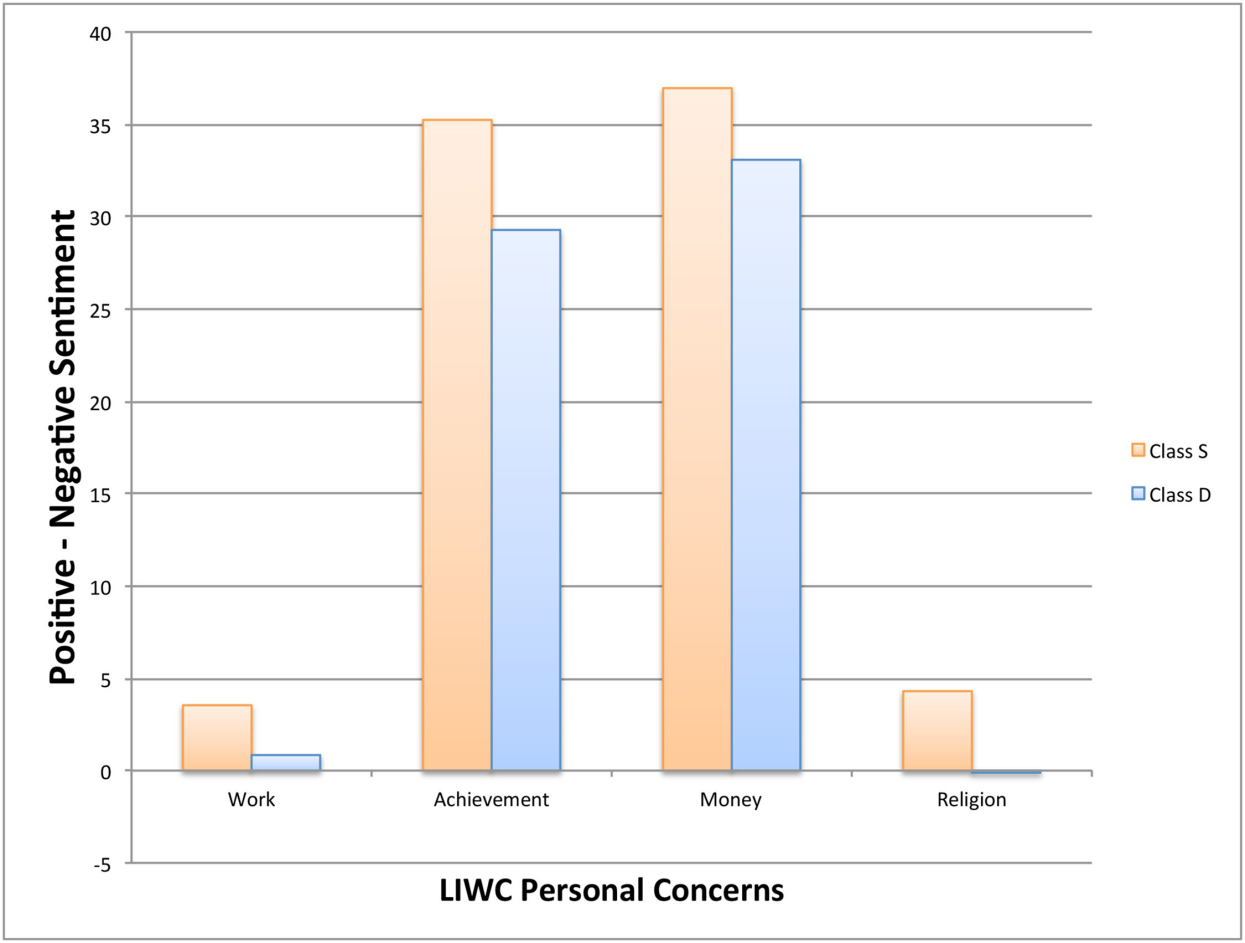
Comparison of user classes on sentiment and LIWC personal concerns. X axis: Personal concerns categories. Y axis: % of positive sentiment tweets − % of negative sentiment tweets. Tweets from Class S users are more positive than negative compared to tweets from Class D users in all categories. The biggest differences are in achievement and religion.

Table 7 compares along key dimensions labeled Personal in LIWC. These are also the dimensions thought to influence life satisfaction (e.g. Lim and Putnam [28], Hadaway [29], and Strine et al. [30]). All categories show significant differences except for Leisure and Home. Money, Religion, and Death show more than 10% difference. Class S more often uses words related to Money and Religion while Class D uses words related to Death more often. Overlaying sentiment analysis on the four categories with a minimum of 5% difference (excluding Death which has a negative loading) we get the comparisons in Fig 3. Interestingly, Class S is more positive on all categories.

**Table 7:** LIWC Personal Concerns. Class S and Class D users differ significantly (Bonferroni-corrected p<0.05) in all except Leisure and Home. Significant differences are in italics, differences > 10% are in bold.

Categories	Example Words	Class S Avg.	Class D Avg.	Sig.	S—D (%)
*Work*	*Job, majors, xerox*	*226.7*	*215.2*	*.0001*	*5.1*
*Achievement*	*Earn, hero, win*	*266.6*	*252.2*	*.0000*	*5.4*
Leisure	Cook, chat, movie	295.0	287.3	.4706	2.6
Home	Apartment, family	80.3	81.1	.0022	-1.0
***Money***	***Audit, cash, owe***	***92.4***	***81.9***	***.0000***	***11.3***
***Religion***	***Altar, church***	***107.3***	***89.0***	***.0000***	***17.0***
***Death***	***Bury, coffin, kill***	***38.0***	***43.9***	***.0000***	***-15.6***

#### 3.2.5 Differences in PERMA categories

Table 8 compares classes along the ten PERMA categories (for the 5 elements of ‘well-being’). Differences in the negative categories are strong (larger than 10%), consistent and in the right direction: Class S exhibits lower frequencies than Class D. For positive categories, Class S has higher frequencies on two, engagement and meaning. But for positive emotion, positive relationships and achievement, Class S has lower frequencies compared to Class D and the differences in the later two are above 5%. When compared to LIWC results, we note that with both lexicons Class S uses less positive emotion than Class D (though the difference is less than 5% in either case). However, with LIWC achievement, usage in Class S is higher than in Class D (7.1%). This conflicts with the PERMA result (Class S < Class D by 7.2%). The lexicons differ, LIWC has 186 achievement words and PERMA has 129 positive achievement words with only 46 overlap. A thorough analysis is required to look at the semantic differences between the two lexicons which should provide further clarification.

**Table 8:** PERMA. Class S and Class D users differ significantly (Bonferroni-corrected p<0.05) in all except Engagement. Significant differences are in italics, differences > 10% are in bold.

Categories	Example Words	Class S Avg.	Class D Avg.	Sig.	S—D (%)
*Positive Emotion*	*satisfied, cheerful*	*83.4*	*86.3*	*.0000*	*-3.5*
***Neg. Emotion***	***suicidal, crying***	***53.4***	***64.4***	***.0000***	***-20.5***
Engagement	careful, devoted	14.7	14.0	.6638	5.1
***Disengagement***	***laziness, weariness***	***15.6***	***17.5***	***.0000***	***-12.5***
*Pos. Relationships*	*neighbors, friendship*	*174.6*	*191.5*	*.0000*	*-9.7*
***Neg. Relationships***	***hate, envy***	***44.0***	***52.6***	***.0000***	***-19.6***
*Meaning*	*represent, religious*	*62.8*	*58.7*	*.0000*	*6.5*
***Lack of meaning***	***looser, vague***	***1.9***	***2.2***	***.0000***	***-17.4***
*Achievement*	*adorned, merit*	*26.3*	*28.2*	*.0000*	*-7.2*
***Lack of Achievement***	***subpar, beaten***	***2.6***	***2.8***	***.0000***	***-10.9***

#### 3.2.6 Summary

This comparison relies primarily on features extracted using LIWC software. We find some differences between Class S and Class D in Twitter metadata features such as in active life span, the number of hashtags and URLs. But there are no differences in the numbers of followers and followings. Language differences exist, most interesting in the numbers of swear words and exclamation marks. In Psychological processes, we find several differences, especially in writings on sexuality, communications of sadness, anger, overall negative emotion and discrepancy (somewhat indicative of aspirations for the future). Overlaying sentiment analysis we find for example Class D is more negative in posts about health, biological processes and sex. In LIWC’s Personal concerns we find most differences in money, religion and death. Again overlaying sentiment analysis we find Class S is more positive about money and religion. We also find that Class D is more negative in all the 5 PERMA elements of “well-being”. The results are more mixed in the positive categories of PERMA. Comparisons in this section were ‘static’ in that time of post is not considered. Temporal analysis is presented next.

### 3.3 Differences between Users in Temporal Characteristics

In 1999 Lewinsohn et al. [24] studied how life satisfaction and psychosocial variables relate over time. They surveyed a group of individuals at two time points, 8 months apart, on life satisfaction, depression, social support, personality, health etc. They then analyzed 4 groups: those with high (or low) LS ratings in both surveys and the two groups where LS ratings changed from one to the other. They found for example that people with low life satisfaction were more likely to have depression later. Inspired by this work we present two types of temporal analyses. The first is of users who always post LS tweets of one kind only, either satisfaction or dissatisfaction. The second analysis is of users who change their life assessment at some point, tweeting first a satisfaction (or dissatisfaction post) and later a post of the opposite status. We examine expressions of psychosocial features before, around and after the time of posting the LS tweet.

Lewinsohn et al. [24] explored depression, social support and social interaction, pleasant activities, cognitions (e.g. irrational beliefs, expectancies of positive and negative outcomes), stress, personalities, health, and demographic variables like gender, age and marital status. Of these we are able to map social support to ‘*Friends and Family*’ category in LIWC, pleasant activities to ‘*Leisure category*’ in LIWC and LIWC has ‘*Health*’. We used a lexical approach to track Depression (with the words ‘*depressing*,’ ‘*depress*,’ and ‘*depression*’). We also consider LIWC categories that were not in Lewinsohn et al. [24]. These are ‘*Anger*’, ‘*Anxiety*’, ‘*Death*’, ‘*Sadness*’, ‘*Home*’, ‘*Money*’, ‘*Religion*’, and ‘*Work*’. Table 9 lists our categories. The table also shows categories (such as ‘*Health*’) differentiated using sentiment analysis.

**Table 9:** Psychosocial Categories. +: Wildcard matching (E.g. ‘*kill*’ can match ‘*killed*,’ ‘*killing*,’ etc.)

Category	Example Words	Lexicon Size
Anger	Hate, kill, annoyed	185+
Anxiety	Worried, fearful, nervous	91+
Death	Bury, coffin, kill	63+
Depression	Depress, depression	2+
Sadness	Crying, grief, sad	101+
Health (Pos)	Health, healthy	234+
Health (Neg)	Clinic, flu, pill	234+
Home (Pos)	Apartment, kitchen, family	93+
Home (Neg)	Apartment, kitchen, family	93+
Leisure (Pos)	Cook, chat, movie	228+
Leisure (Neg)	Cook, chat, movie	228+
Money (Pos)	Audit, cash, owe	173+
Money (Neg)	Audit, cash, owe	173+
Religion (Pos)	Altar, church, mosque	160+
Religion (Neg)	Altar, church, mosque	160+
Social Support (Pos)	Daughter, husband, friend	102+
Social Support (Neg)	Daughter, husband, friend	102+
Work (Pos)	Job, majors, xerox	326+
Work (Neg)	Job, majors, xerox	326+

We studied 4 user groups (1,278 users in each): S (always posted life satisfaction), D (always posted dissatisfaction), and those who changed status, S = >D and D = >S at some point in time with the obvious interpretations. Criteria for selection include: the user should have at least 10 tweets totally and the user account should be at least 300 days old prior to posting their LS tweet. Additionally for S = >D and D = >S, the interval between change of status from S (or D) to D (or S) should be at least 30 days.

**Fig 4:**
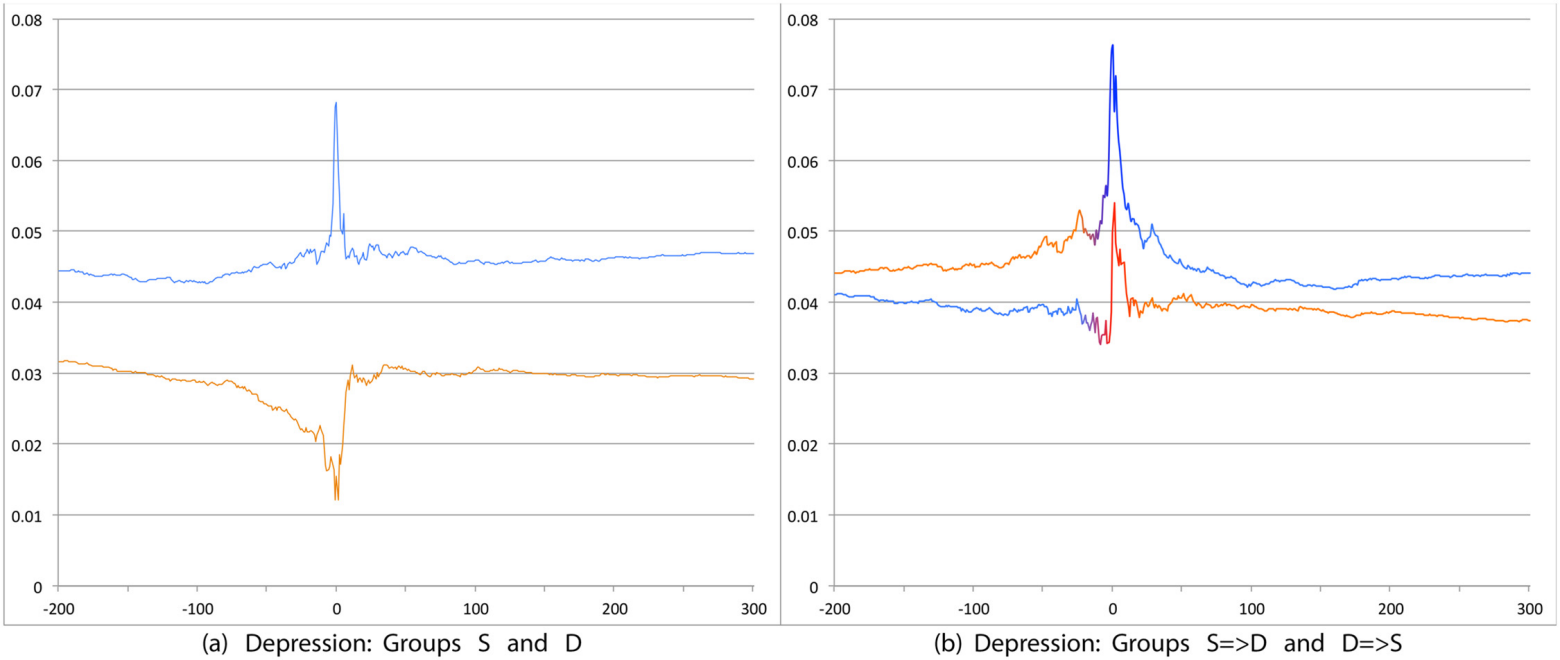
Prevalence of tweets about Depression. X axis day -200 to day +300 around Day 0. Y axis: Prevalence of tweets about Depression calculated using Eqs (1) and (2). The left panel shows trends for S (orange line) and D (blue line) groups while the right panel shows trends for S = >D (orange to blue line) and D = >S (blue to orange) groups.

We compared groups using tweet prevalence plots centered on the notion of a ‘Day 0’ defined specific to each user as follows. For S (and D) this is the date on which the user posted the first satisfaction (dissatisfaction) tweet. For S = >D and D = >S, this is the date when the user posted the first LS tweet contradicting their previous status. (See Fig 4 as an example prevalence plot for the psychosocial variable Depression). For points on the X axis to the right of 0 the prevalence score is calculated by accumulating tweets from Day 0. For points on the X axis to the left of 0 we accumulate tweets from that day to Day 0. Thus Day 0 is a fulcrum point for combining trends across a set of users. We calculate prevalence percentages at different days for each combination of psychosocial variable and user group. Two examples are given below.

$$\begin{array}{c} Prevalence\left(S,anger,+100\right)\\ =\frac{\text{\# first person tweets conveying anger from group S between days 0 to +100}}{\text{\# first person tweets from group S between days 0 to +100}}*100 \end{array}$$ (1)

$$\begin{array}{c} Prevalence\left(D,anxiety,-200\right)\\ =\frac{\text{\# first person tweets conveying anxiety from group D between days -200 to 0}}{\text{\# first person tweets from group S between days -200 to 0}}*100 \end{array}$$ (2)

**Fig 5:**
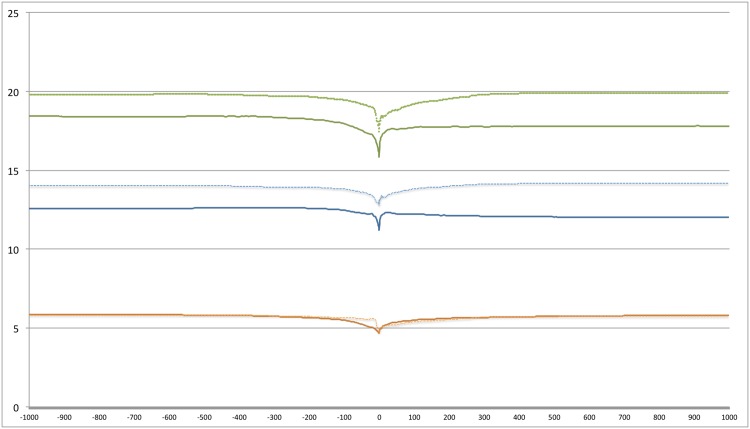
Tweet distribution around ‘Day 0’. X axis: Time in days around day ‘0’. Y axis: Percentages calculated as per Eqs (1) and (2). The only difference is that the accumulations are across multiple categories. Orange, blue and green represent positive, negative and all categories respectively. Continuous lines represent group S while dashed lines represent group D.

We start with Fig 5 presenting three summary prevalence trends. These summaries are across a) all positive psychosocial variables b) all negative psychosocial variables and c) all psychosocial categories. See Table 9 for these categories. About 20% of the first person tweets posted by Group D tends to be about the psychosocial categories. (The fact that there are more tweets on the negative side than positive is an artifact since we are considering more negative than positive categories.) Focusing on the negative categories (blue lines), we note that these tweets are more prevalent from Class D than Class S. This also appears to account for differences between the two groups in totals (green lines). Overall the trend lines are steady except for a dip around Day 0, more so for negative than for positive categories. This is not an outcome of the accumulation process since we observe a dip on Day 0, even if a different day is chosen as the fulcrum point for accumulation. It appears that on and just around the day that a life satisfaction post is made the tendency to post on these psychosocial categories reduces.

**Fig 6:**
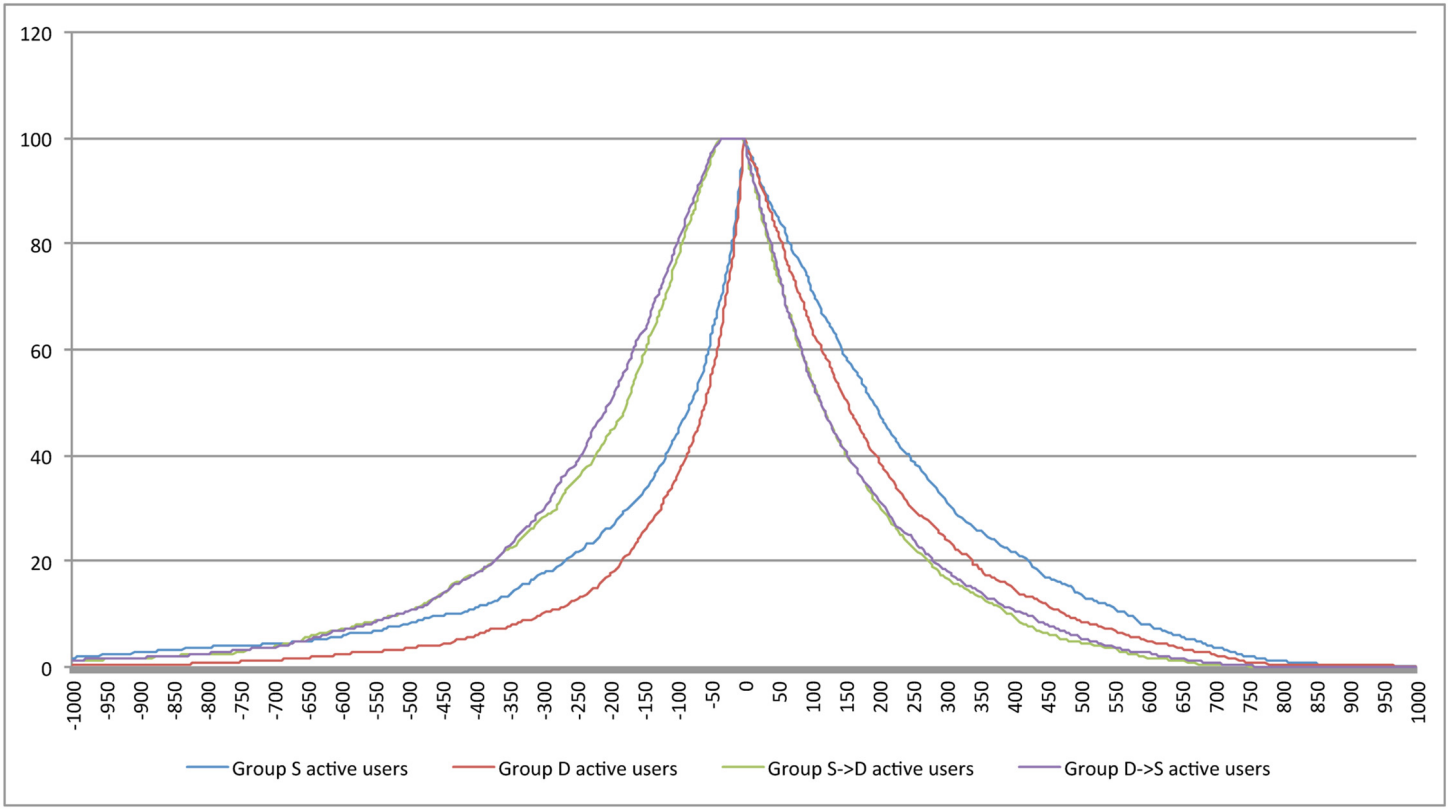
User attrition rates for groups. X axis: day -1000 to day +1000 relative to Day 0. Y axis: Percentage of Day 0 users still active. For example at least 50% of the 1,278 S users are active between day -80 and day +150.

The X-axis in Fig 5 spans from day -1000 to day +1000. The question to ask is how many users are actually active over this long time span? Active life span of a user is the time between the first and the last tweet. We are assured of 1,278 active users in each group on Day 0 but there will be attrition as we move away from Day 0 in either direction. Attrition rate is important as comparisons involving time points with few users will not be meaningful. Fig 6 shows attrition for each group. For example, 50% of S users are active at -80 days and at +150 days. Table 10 highlights select data points. For example that attrition is faster for S and D compared to the other groups before Day 0 but not so after Day 0. Given attritions we limit our analysis to the range having about a minimum of 20% of the users (about 250 users in each group). Thus we limit analysis to the range Day -200 to Day +300.

**Table 10:** Number of days before and after ‘Day 0’ for different percentages of active users.

Group	Before ‘Day 0’			At ‘Day 0’	After ‘Day 0’		
	20%	50%	75%	100%	75%	50%	20%
S	-270	-80	-20	1,278	90	150	420
D	-185	-60	-20	1,278	70	190	340
S⇒D	-375	-175	-115	1,278	50	115	285
D⇒S	-375	-200	-115	1,278	50	115	285

We first compare S and D groups and then S = >D and D = >S groups. Relevant prevalence plots for the psychosocial categories are Figs 4 (Depression), 7 (Death), 8 (Sadness), 9 (Anger), 10 (Anxiety), 11 (Health), 12 (Home) and 13 (Religion). In these the X-axis represents days and the Y-axis represents a percentage as calculated by Eqs (1) and (2). Blue lines stand for the D group and orange lines for S group. Color transitions in the S = >D and D = >S graphs indicate change in LS status. Plots have two panels each, one comparing S and D and the other comparing S = >D and D = >S groups. Plots for Home, Health and Religion have 4 panels since each category is differentiated by sentiment (e.g., Home(neg) and Home(pos)).

**Fig 7:**
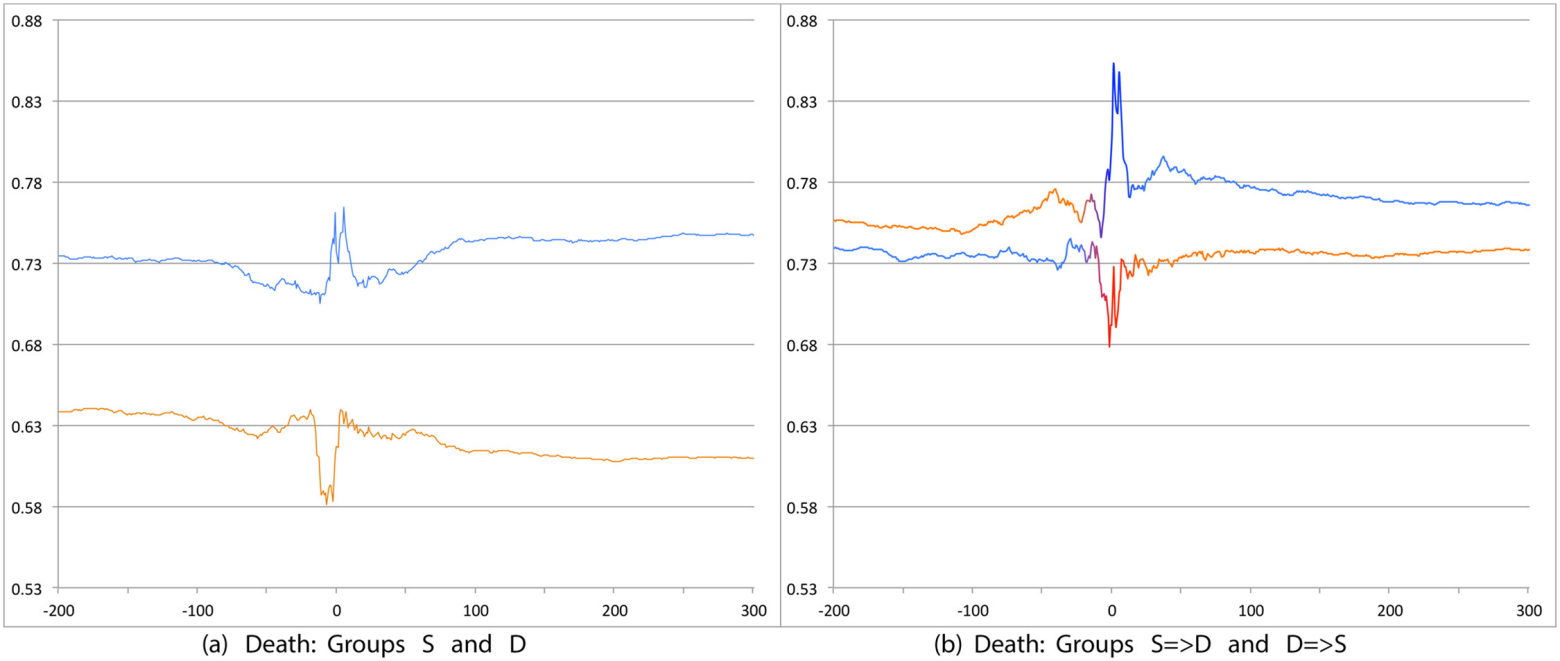
Prevalence of tweets about Death. X axis day -200 to day +300 around Day 0. Y axis: Prevalence of tweets about Death calculated using Eqs (1) and (2). The left panel shows trends for S (orange line) and D (blue line) groups while the right panel shows trends for S = >D (orange to blue line) and D = >S (blue to orange line) groups.

**Fig 8:**
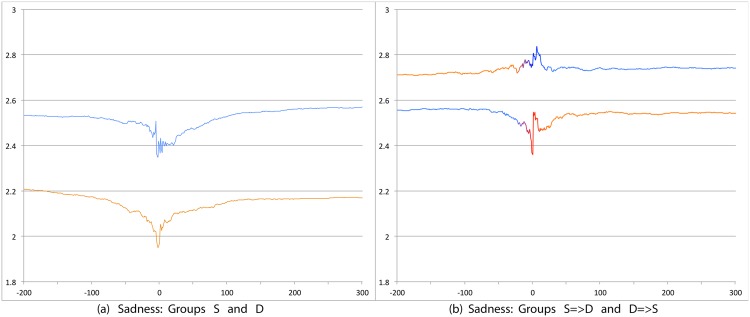
Prevalence of tweets about Sadness. X axis day -200 to day +300 around Day 0. Y axis: Prevalence of tweets about Sadness calculated using Eqs (1) and (2). The left panel shows trends for S (orange line) and D (blue line) groups while the right panel shows trends for S = >D (orange to blue line) and D = >S (blue to orange line) groups.

**Fig 9:**
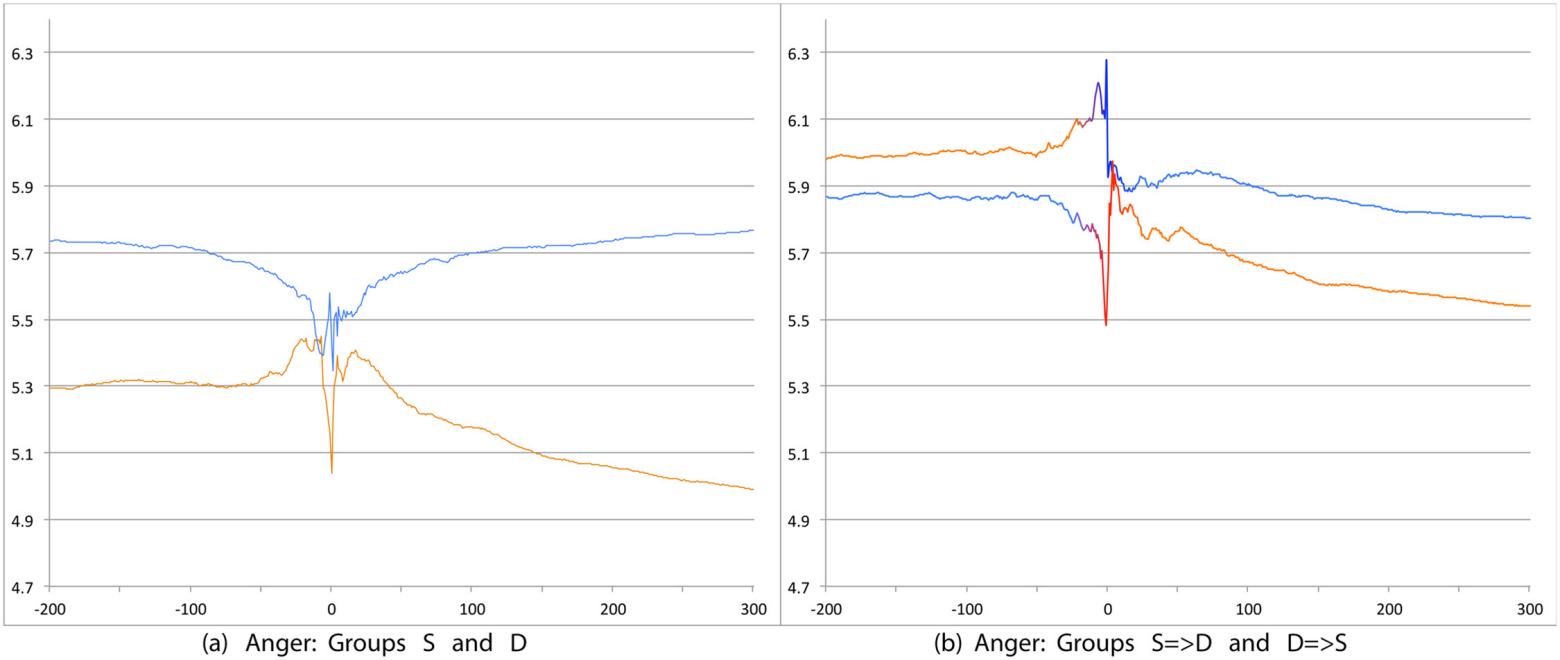
Prevalence of tweets about Anger. X axis day -200 to day +300 around Day 0. Y axis: Prevalence of tweets about Anger calculated using Eqs (1) and (2). The left panel shows trends for S (orange line) and D (blue line) groups while the right panel shows trends for S = >D (orange to blue line) and D = >S (blue to orange line) groups.

**Fig 10:**
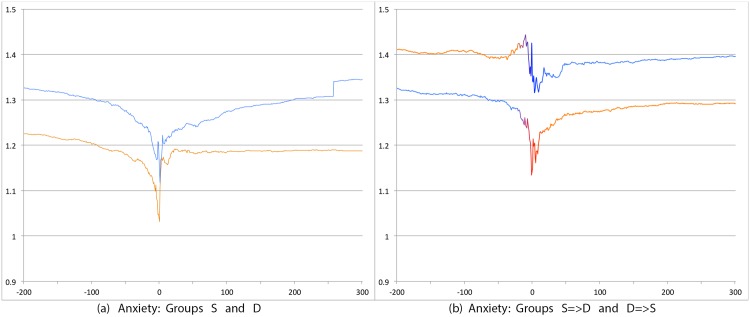
Prevalence of tweets about Anxiety. X axis day -200 to day +300 around Day 0. Y axis: Prevalence of tweets about Anxiety calculated using Eqs (1) and (2). The left panel shows trends for S (orange line) and D (blue line) groups while the right panel shows trends for S = >D (orange to blue line) and D = >S (blue to orange line) groups.

**Fig 11:**
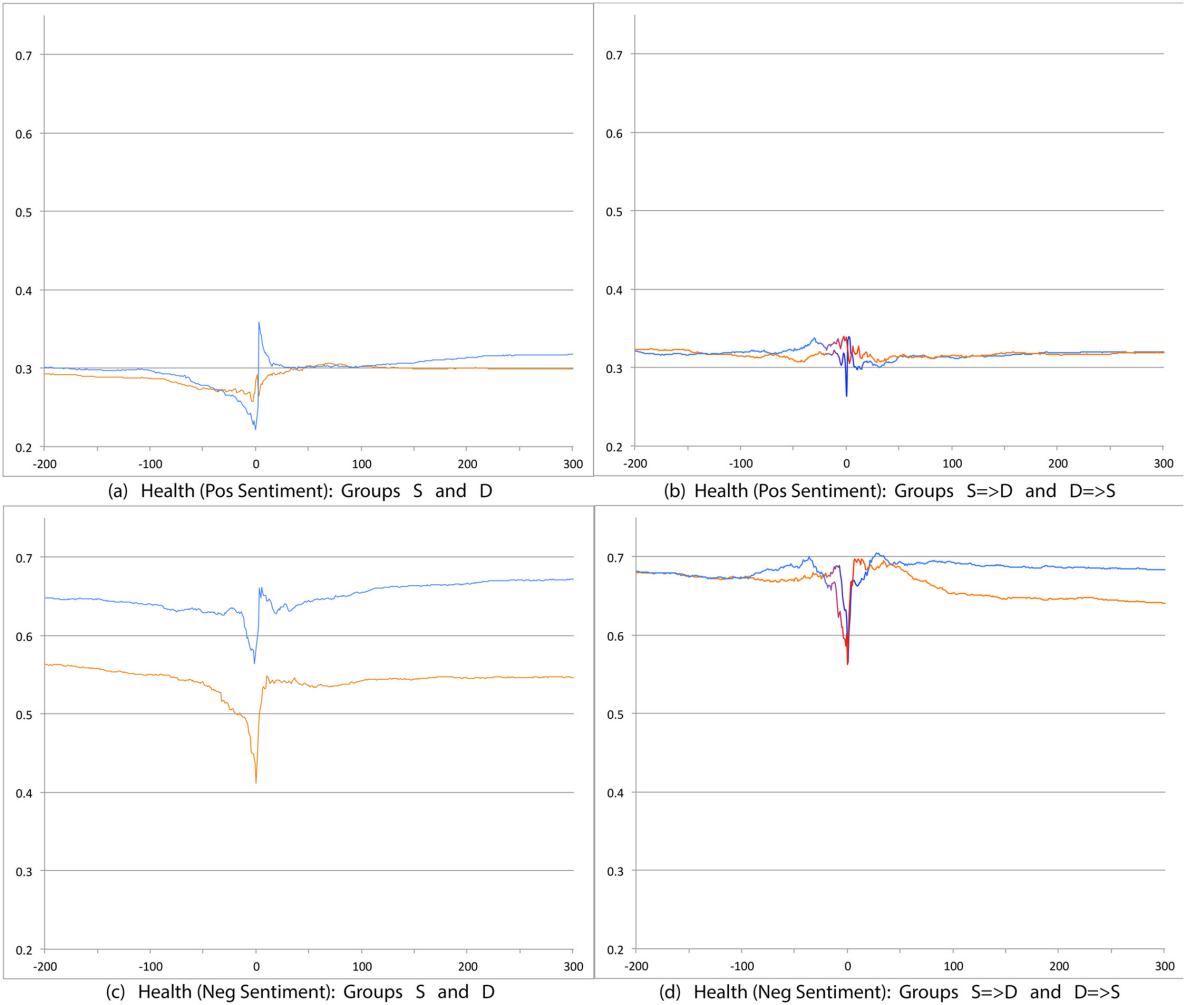
Prevalence of tweets about Health. X axis day -200 to day +300 around Day 0. Y axis: Prevalence of tweets about Health calculated using Eqs (1) and (2). The top panels are trends for tweets indicating positive sentiment about Health. The bottom panels are for negative sentiment about Health. The left panels shows trends for S (orange line) and D (blue line) groups while the right panels shows trends for S = >D (orange to blue line) and D = >S (blue to orange line) groups.

**Fig 12:**
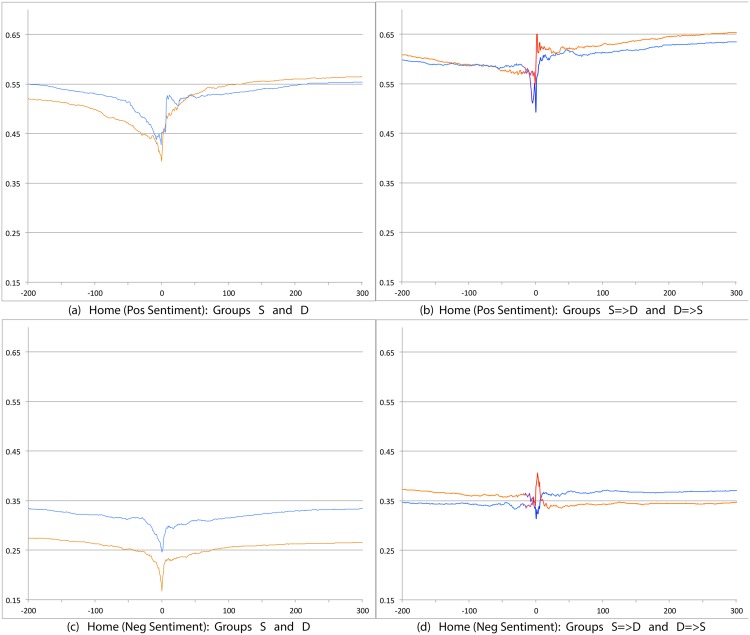
Prevalence of tweets about Home. X axis day -200 to day +300 around Day 0. Y axis: Prevalence of tweets about Home calculated using Eqs (1) and (2). The top panels are trends for tweets indicating positive sentiment about Home. The bottom panels are for negative sentiment about Home. The left panels shows trends for S (orange line) and D (blue line) groups while the right panels shows trends for S = >D (orange to blue line) and D = >S (blue to orange line) groups.

**Fig 13:**
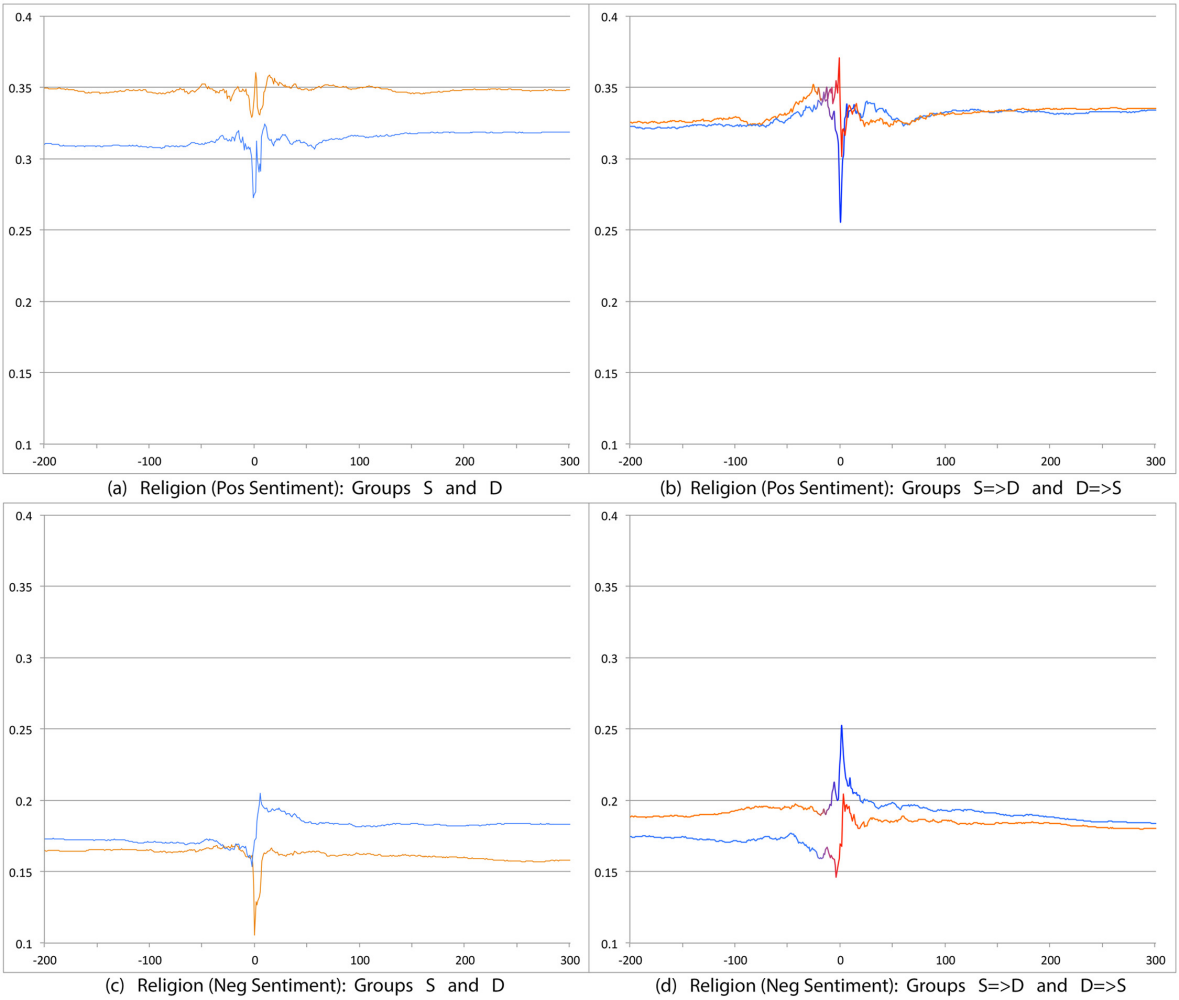
Prevalence of tweets about Religion. X axis day -200 to day +300 around Day 0. Y axis: Prevalence of tweets about Religion calculated using Eqs (1) and (2). The top panels are trends for tweets indicating positive sentiment about Religion. The bottom panels are for negative sentiment about religion. The left panels shows trends for S (orange line) and D (blue line) groups while the right panels shows trends for S = >D (orange to blue line) and D = >S (blue to orange line) groups.

#### 3.3.1 S Users versus D Users

Consistent with intuitive expectations group D posts more throughout the timeline than group S on the negative psychosocial categories: Depression, Death, Sadness, Health(neg), Home(neg), Work(neg), Social Support(neg). This is also the case with Anger, Anxiety and Religion(neg), though the trends for S and D overlap just slightly around Day 0. D users also post more than S for Leisure(neg), though the difference is small. In general all of these graphs show dips around Day 0. The notable exceptions are for Death and Depression where the trends for S show a dip and the trend lines for D actually go up around Day 0.

S and D groups do not show clear or consistent differences on positive variables. The one observation that stands out is that S users post more than D throughout on Religion(pos). As mentioned earlier, D users post more than S on Religion(neg).

Table 11 summarizes these trends ignoring the region around Day 0. The 4th column indicates whether the pattern of difference observed is as intuitively expected or surprising. Inconsistent differences are seen only in a couple of trends. One pattern (for (Leisure(pos)) is marked ‘Unclear;’ even though the trend is consistent its interpretation is unclear. Overall all the observed differences are as intuitively expected.

**Table 11:** Comparison of groups S and D on psychosocial categories. Inconsistent: trends before and after Day 0 are not the same >: S has significantly more psychosocial tweets than D <: S has significantly fewer psychosocial tweets than D.

Category	Before Day 0 S vs. D	After Day 0 S vs. D	Pattern
Anger	<	<	As expected
Anxiety	<	<	As expected
Death	<	<	As expected
Depression	<	<	As expected
Sadness	<	<	As expected
Health (Pos)	<	<	Surprising
Health (Neg)	<	<	As expected
Home (Pos)	<	>	Inconsistent
Home (Neg)	<	<	As expected
Leisure (Pos)	<	<	Unclear
Leisure (Neg)	<	<	As expected
Money (Pos)	>	>	As expected
Money (Neg)	<	<	As expected
Religion (Pos)	>	>	As expected
Religion (Neg)	<	<	As expected
Social Support (Pos)	>	>	As expected
Social Support (Neg)	<	<	As expected
Work (Pos)	<	>	Inconsistent
Work (Neg)	<	<	As expected

#### 3.3.2 S = >D users versus D = >S users

Overall differences were smaller between these two user groups compared to between S and D. Notable differences were found in the pure negative categories: Anger, Anxiety, Sadness, Death, and then Depression. Interestingly, in each of these negative categories those who start off satisfied and change to dissatisfied post more than those who start dissatisfied and change to satisfied. Noteworthy too is that tweet prevalence from S = >D users in these 5 negative categories were each higher or equal to those from the stable dissatisfied D users; these in turn were higher than for the stable satisfied S users! That is, the group changing from satisfied to dissatisfied looks very different from the one that stays satisfied throughout. While these observations are made at the group level, an idea to explore in future research is whether tweet frequency in these categories may be used to predict which user is likely to change of expression from life satisfaction to dissatisfaction. Trends in the other categories were unremarkable because of small differences (even if significant) except for Religion(neg). Here we see S = >D users posting more than D = >S, especially before Day 0. Table 12 summarizes these trends ignoring the region around Day 0. The last column of the table is limited to noting consistency and inconsistency as we have few intuitive expectations of patterns of differences between these two groups.

**Table 12:** Comparison of groups S⇒D and D⇒S on psychosocial categories. >: S⇒D has significantly more psychosocial tweets than D⇒S <: S⇒D has significantly fewer psychosocial tweets than D⇒S =: Groups has similar number of PV tweet in the figure (lines have overlap, even though it may be statistically significant).

Category	Before ‘Day 0’ S⇒D vs. D⇒S	After ‘Day 0’ S⇒D vs. D⇒S	Pattern
Anger	>	>	Consistent
Anxiety	>	>	Consistent
Death	>	>	Consistent
Depression	>	>	Consistent
Sadness	>	>	Consistent
Health (Pos)	>	<	Inconsistent
Health (Neg)	=	>	Inconsistent
Home (Pos)	>	<	Inconsistent
Home (Neg)	>	>	Consistent
Leisure (Pos)	>	>	Consistent
Leisure (Neg)	>	>	Consistent
Money (Pos)	=	<	Inconsistent
Money (Neg)	=	>	Inconsistent
Religion (Pos)	>	=	Inconsistent
Religion (Neg)	>	>	Consistent
Social Support (Pos)	=	<	Inconsistent
Social Support (Neg)	>	>	Consistent
Work (Pos)	>	<	Inconsistent
Work (Neg)	>	>	Consistent

#### 3.3.3 Summary

Overall temporal patterns related to psychosocial categories show greater differences between S and D user postings compared to S = >D versus D = >S users. Death, Depression, Anxiety, Anger and Sadness categories yield strong, intuitively consistent results. The remaining categories, especially on the negative side also provide intuitive and consistent results. The least interesting results are from comparisons on the positive variables. The one exception is Religion.

## 4 Discussion

We present results from the first ever surveillance of life satisfaction expressions on Twitter. The research complements recent investigations of affect expressions [5–13] (termed happiness by the authors) in that both affect and life satisfaction are components of subjective well-being. While affect has been studied in several papers, life satisfaction has received far less attention especially with Twitter data [14–17].

We build on recent research where we develop and test a strategy for surveillance of life satisfaction expressions [22]. Our strategy is unique in that it is founded on a well-respected and widely used survey of life satisfaction (SWLS) and it emphasizes both precision and recall. In this regard our strategy stands apart from general social media surveillance research (e.g., for flu [31], depression [32], etc.). Our previous paper paves the way for deriving surveillance strategies from other surveys. In this paper we apply the surveillance strategy to a two-year stretch of Twitter data. This data is intentionally limited to first person tweets that are written in the present tense so as to mimic self-ratings in the SWLS survey as much as possible.

Our current results provide additional validity to our surveillance method. In the previous paper, we assessed the method using annotated data and by comparing with more standard approaches. In this paper a survey of select Twitter users using the same SWLS survey produced results consistent with results from the surveillance strategy. This portion of our research also indicates the extreme difficulty of conducting surveys with specific Twitter users.

The temporal prevalence trends extracted from the two-year Twitter stream indicate that expressions of life satisfaction are immune to current events (political, seasonal, disasters etc.) and show only random fluctuations. This is in stark contrast to the findings of Dodds et al., [9] where they show that the trend in happiness (affect) is influenced strongly by current events and illustrate this with many examples. These two observations across papers are jointly consistent with the overall definitions of life satisfaction (as a cognitive and long-term assessment of one’s own life), and affect (positive and negative emotion felt daily due to various reasons). Thus we expect and we see stability in life satisfaction expressions on Twitter and we expect and we see external events influencing trends in affect in Dodds et al., research [9]. The fact that the trends are consistent with definitions further validate our methods. Note that stability in life satisfaction does not imply that a person may not change his/her assessment. It only means that life satisfaction assessments are stable compared to affect.

When we compare Twitter users expressing satisfaction with those expressing dissatisfaction we do not find differences in followers and followings. Instead we find linguistic differences such as in the usage of urls, hashtags, personal pronouns, first person singular structures. More interesting observations are on differences in the use of swear words, in conveying negative emotion, anger, sadness and sexuality, etc. These are more frequent amongst users expressing dissatisfaction. There are also differences in prevalence of posts on money, religion and death.

We can also compare our results with those in Table 2 of Schwartz et al. [17]. Despite differences between the two studies (discussed before), we find interesting consistencies. We have also tested 15 of the 20 features listed on their Table 2. We are in strong agreement in 7 of these 15 features, with differences between satisfied and dissatisfied tweeters larger than 10% and in the right direction. These are money, negative relationships, sexual terms, swear words, negative emotion, anger, disengagement. For example we find Class S posts more than Class D in money and this feature is positively correlated with life satisfaction in [17]. Also Class D posts more sexual terms than Class S and this feature is negatively correlated in [17]. Additionally we note through our sentiment overlays that Class S is also more positive in the money posts and Class D is more negative in the sexual posts. If we consider a difference threshold of 5% then we have consistent results for another 3 features: work, achievement and body. A difference is that they find positive emotion to be strongly correlated with life satisfaction. In contrast, we find that Class S posts slightly less than Class D (less than 5% difference). As said earlier both groups are consistent in findings regarding negative emotion. We postulate that our results may differ because of a combination of two points. First the individuals whose writings we study are the ones who post on life satisfaction. Second, it is recognized in several systems of thought (from popular self-help (e.g., [33]) to older philosophical systems (e.g, [34])) that emotional stability leads to greater satisfaction in life.

Temporal analysis of our Twitter data provide results that can be intuitively appreciated. For example, users expressing life dissatisfaction post more tweets throughout the timeline on all clearly negative categories considered, death, anxiety, depression, sadness and anger compared to users expressing life satisfaction. Those who change from satisfaction to dissatisfaction also post more on these categories than those who change the other way. Overall, the results for satisfied users compared to dissatisfied users are different in the intuitively correct direction for almost all of the negative categories. Differences for the positive categories are not remarkable with the exception of Religion(pos).

Our results are also consistent with observations and conclusions made in life satisfaction research from sociology and psychology. We highlight some parallels. Twitter users who express life satisfaction post more positive tweets and fewer negative tweets on religion compared to users expressing life dissatisfaction. The link between religiosity and life satisfaction is well documented in sociology and the current focus appears to be more on understanding the mechanism by which the two relate (Lim and Putnam [28], Ferriss [35], Greeley and Hout [36], Hadaway [29], Inglehart [37]). It is not surprising that in psychology and psychiatry there is a strong line of inquiry on the link between life dissatisfaction and depression. Studies indicate that the two tend to exhibit concurrently (Heli Koivumaa-Honkanen et al. [38]). Interestingly the authors also suggest that the life satisfaction scale itself may be used to assess the risk of developing depression in the future [38]. Guney et al. [39] also find negative and significant correlations between life satisfaction on one side and depression and anxiety on the other side. Our Twitter observations are consistent with these findings.

There are also papers examining various angles such as correlations between poor health and life satisfaction, about life dissatisfaction indicating an elevated risk for adverse health, on the link between declining social support and declining life satisfaction (e.g., Strine et al. [30]). Similar observations are in (Palmore and Luikart [40], Wan et al. [41]). Again our results are generally consistent. We find that Twitter users expressing life satisfaction make fewer negative posts about social support and about health than users expressing life dissatisfaction.

Probably the most important general finding from our study is that we can gather meaningful observations about life satisfaction from Twitter, a large, global social network. The observations are consistent with the definition of life satisfaction in the social sciences; the observations clearly differentiate between life satisfaction and affect, key components of subjective well being and they are consistent with findings in social science research on associated factors. Our results also provide additional, albeit indirect, validation for our surveillance tool. Several directions are open for future work. We have not considered gradations in satisfaction levels and leave this complex goal for future research. We could explore a prediction model to predict change from satisfied to dissatisfied using the features explored and additional ones. We could also study the Twitter social networks of different user groups for further insight.

## Methods

The study is approved by university of Iowa IRB. IRB ID #: 201307803, PI: Chao Yang, Title: Expressions of Satisfaction With Life by Twitter Users.

### 4.1 Overview of Surveillance Method

We start in a principled way with a highly reputed self-assessment scale in psychology called the Satisfaction With Life Scale (SWLS) designed in 1985 by Diener [18] et al. The scale has been cited more than 9,800 times and used in many areas, including studies in psychology [42] and social media [15] (but not in the manner we propose). The scale has five statements as shown in Table 13. A respondent is asked to self-rate for each statement on a scale from 1 to 7 (Strongly Disagree (1); Disagree; Slightly Disagree; Neither Agree or Disagree; Slightly Agree; Agree; Strongly Agree (7)). Notice that the SWLS survey deliberately steers away from specific criteria such as career, home, family, etc. Our goal is to find tweets that can be seen as valid responses to the survey. Those tweets are the life satisfaction (LS) tweets of interest.

**Table 13:** Diener et al.’s [18] Satisfaction With Life Scale.

Statement
1. In most ways my life is close to my ideal.
2. The conditions of my life are excellent.
3. I am satisfied with life.
4. So far I have gotten the important things I want in life.
5. If I could live my life over, I would change almost nothing

Given the many possible ways for expressing life satisfaction assessments in tweets, we developed a process that takes statements in the scale and generalizes them into templates. Each template is then transformed into a set of retrieval strategies. Finally, we use an information retrieval system to retrieve the LS tweets from a daily Tweet collection using these search queries. In this manner, we transformed the survey into a Twitter surveillance strategy. While details are in [22], we briefly summarize the process here. Table 14 is used to illustrate this process with one statement from the SWLS.

**Table 14:** Illustration of Methodology.

**Step 1.** Starting Point: A statement from SWLS Ex: ‘*I am satisfied with life*’ (SWLS Statement 3)
**Step 2.** Obtain alternate statements from crowdworkers Ex: ‘*my life is peachy*’, ‘*I am totally loving my life*’
**Step 3.** Convert statements to general templates Ex: ‘*my life is [peachy]*’ Because of the generalized template structure we also consider many statements such as: ‘*my life is great*’; ‘*my life is wonderful*’; ‘*my life is fabulous*’ etc. These expansions are done using a lexicon of synonyms.
**Step 4.** Build retrieval strategies. Multiple strategies are generated for each template. These are built by combining one option from each of 1) and 2) below. The strictest strategy is when we select 0 for both. 1) Allow 0, 1, 2, or 3 words in between words of the template. 2) Allow 0, 1, 2, or 3 additional words before and after the template Ex: The tweet ‘*My life is really peachy*’ will be retrieved (1 word in between and no additional words before or after) Ex: The tweet ‘*My life is truly peachy for ever*’ will be retrieved (1 word in between and up to 2 words before and after).
**Step 5.** Run the retrieval strategies on the tweet dataset to retrieve life satisfaction tweets.
**Step 6.** Filter retrieved tweets.

Staring with an SWLS survey statement (Step 1) we first obtained an initial set of essentially equivalent statements through crowdsourcing with MTurk (Step 2). For example the sentence ‘*my life is peachy*’ is synonymous to the SWLS statement 3.

Next, we manually generalized the statements into templates (Step 3); each template supports many equivalent expressions. Bracketed words in a template may be substituted by a variety of synonyms from a lexicon or a dictionary (e.g., happy, delighted, content are synonymous in this context). We built two sets of templates to retrieve tweets expressing life satisfaction versus life dissatisfaction. Dissatisfaction templates have their own synonym sets e.g., ‘*sad, depressing, miserable*’

Then we built a set of 16 retrieval strategies (search queries) from each template (Step 4). The strategies differ along 2 dimensions: i) the number of intervening words allowed in a template (P) and ii) the number of words allowed before or after a text segment that satisfies the template (Q). We vary P and Q over (0,1,2,3) which gives us 16 combinations for building retrieval strategies. The strictest strategy is when P and Q are set to 0. Here for example, we would look for tweets saying ‘*my life is peachy*’ (or ‘*my life is fantastic*’ etc.) and exactly that. With P = 2 we also retrieve ‘*life is oh so peachy*’. With Q = 2 we also get tweets stating ‘*For sure life is peachy for me*’. The square brackets indicate that synonyms may be used (‘*wonderful, excellent, peachy*’ etc.). A similar set of strategies is built to obtain the dissatisfaction tweets with appropriate synonyms such as ‘*horrible, horrid, terrible*’. In step 5, we use the retrieval strategies to retrieve life satisfaction tweets from the dataset. Finally in step 6 we use a few filters to a) remove irrelevant tweets and to b) deal with negation. For example the tweet ‘*My life is amazing because of my cat.*’ is not relevant. Similarly tweets in past or future tense (‘*I used to have a happy life*’), referring to third persons (‘*my friend has a great life*’), asking questions (‘*Is my life good?*’), etc. are filtered out. When the satisfaction templates retrieve tweets with negation such as ‘*My life is not perfect*’ and ‘*I am not happy*,’ these are automatically moved to our set of dissatisfaction tweets. Note that ‘*my life is imperfect*’ or ‘*unhappy*’ are captured directly in the dissatisfaction templates. Overall, our template based retrieval strategies emphasize precision while also accommodating a variety of synonymous expressions to enhance recall. Crucially we focus on tweets that are in the first person (FP), so that we have a better chance of finding expressions that approximate self-ratings. This overview is necessarily limited, details are provided in our previous paper [22].

We provide our two-day life satisfaction gold standard dataset (about 7.42 million tweets) as well as a random sample of life satisfaction tweets automatically detected in 2013 (25K for each class) using our method in S1 and S2 Datasets respectively. Due to the restriction of Twitter, we only provide the tweet ID for the life satisfaction irrelevant tweets in the gold standard dataset and the random sample of automatically detected LS tweets. In addition, we provide the lexicon and templates used in our method in S1 Lexicon and S1 Template respectively.

## Supporting Information

S1 Fig Distribution of tweets with the keywords ‘Morning,’ ‘Noon,’ and ‘Evening’ over hours of the day. Peak points for each keyword are as to be expected.
(TIF)

S2 Fig Distribution of tweets with the keywords ‘Breakfast,’ ‘Lunch,’ and ‘Dinner’ over hours of the day. Peak points for each keyword are as to be expected.
(TIF)

S3 FigTrend in postings of life satisfaction tweets over a two-year period.X axis: week. Y axis: Percentage computed as $\frac{\text{\# Class S (or D) tweets in a day}}{\text{\# First Person Tweets in the day}}\times 100\%$.(TIF)

S4 Fig Complementary cumulative distribution function (CCDF) for number of followers on a log-log graph. Yellow represents Class S users and blue represents Class D users. The two groups show unremarkable differences.
(TIF)

S5 Fig Complementary cumulative distribution function (CCDF) for number of followings on a log-log graph. Yellow represents Class S users and blue represents Class D users. The two groups show unremarkable differences.
(TIF)

S1 Dataset Two-day life satisfaction gold standard dataset. (ZIP)

S2 Dataset Random sample of life satisfaction tweets automatically detected in 2013. (ZIP)

S1 Lexicon Lexicon of our template based method. (TXT)

S1 Template Templates of our template based method. (TXT)
