# Supplementary figures and images for: Life Satisfaction and the Pursuit of Happiness on Twitter
Source: PLoS One. 2016 Mar 16;11(3):e0150881. doi: 10.1371/journal.pone.0150881 (PMC4794168; doi:10.1371/journal.pone.0150881)

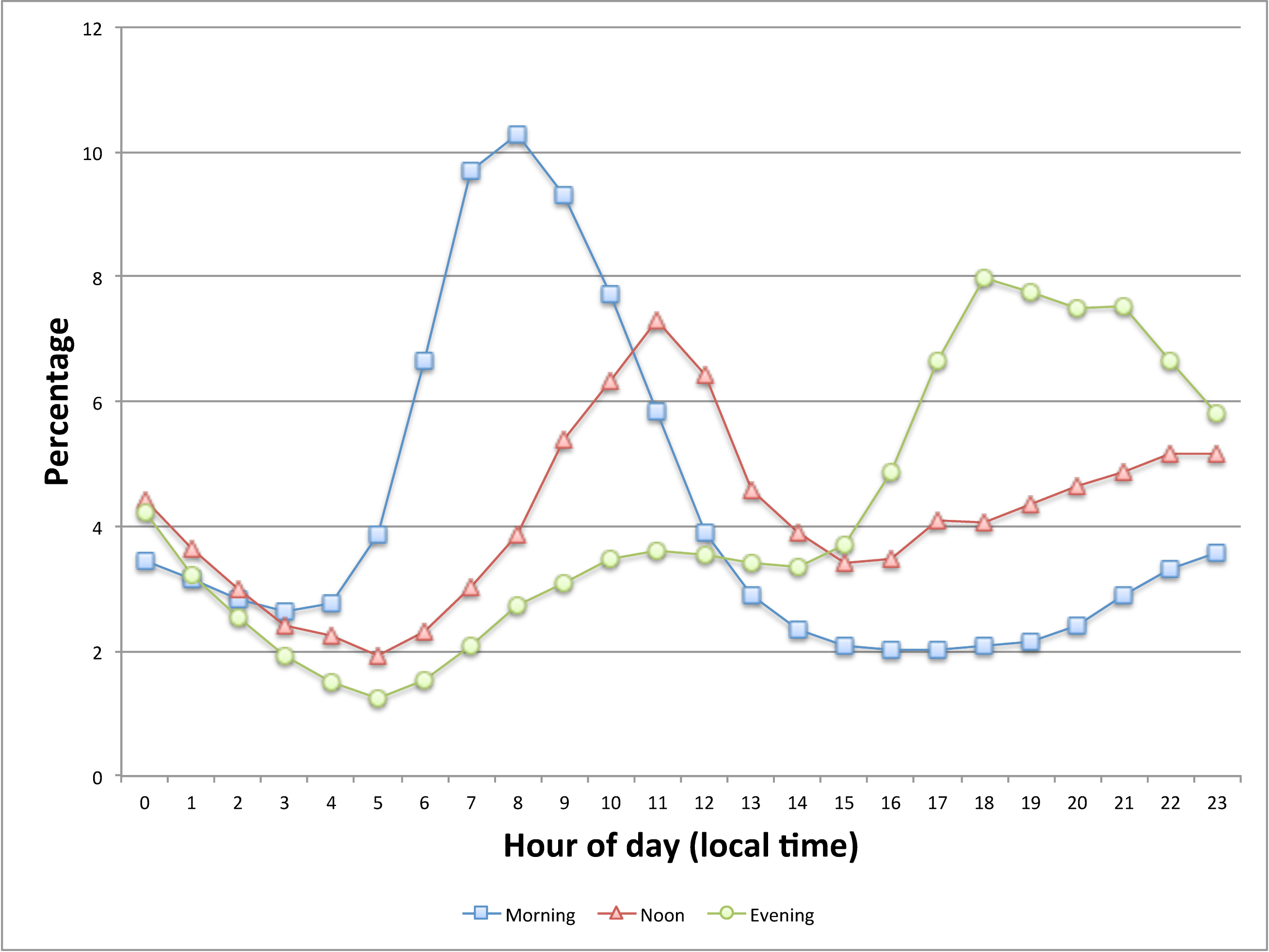

Supplement: S1 Fig — Peak points for each keyword are as to be expected. (TIF) [file pone.0150881.s001.tif]

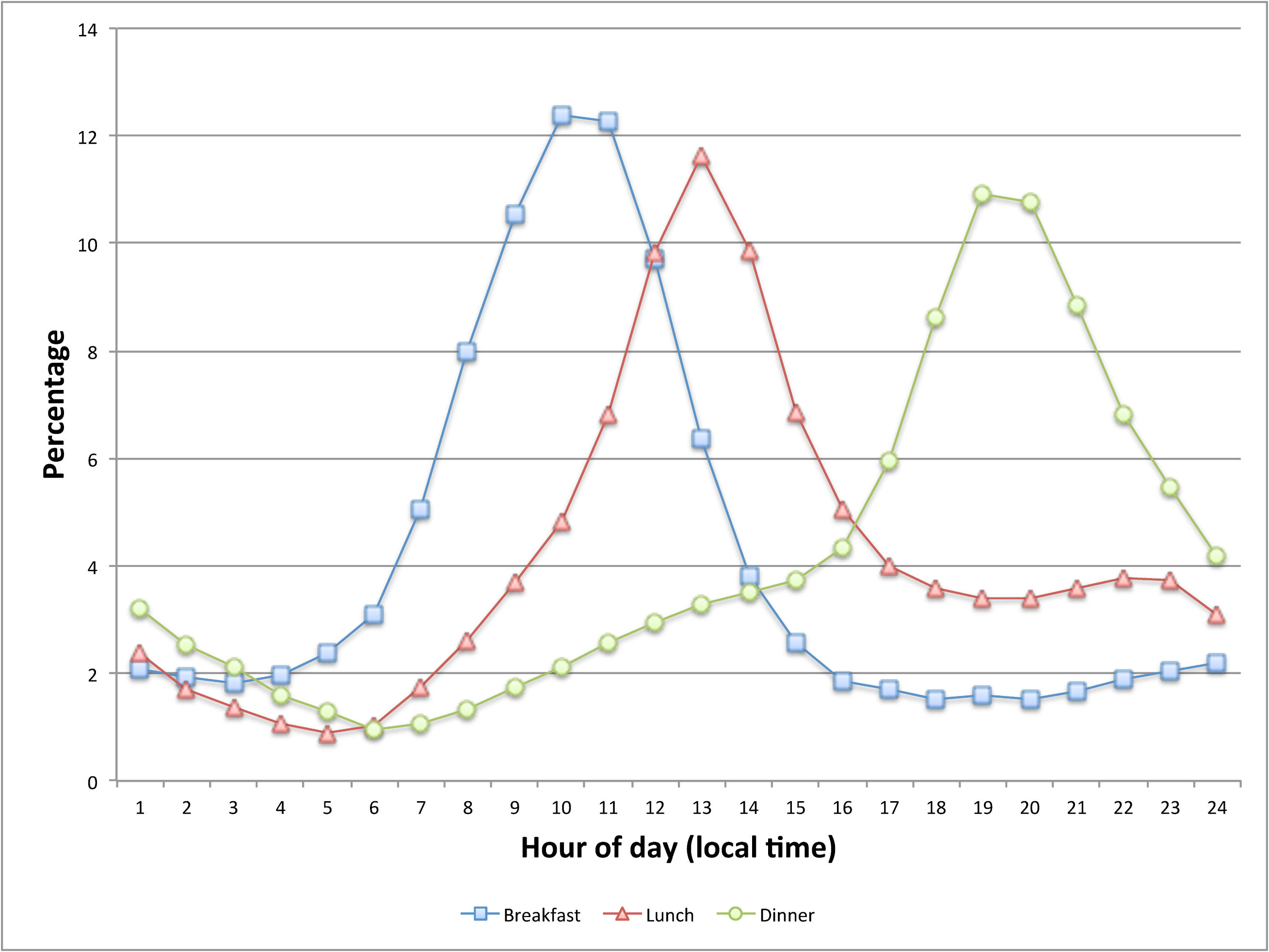

Supplement: S2 Fig — Peak points for each keyword are as to be expected. (TIF) [file pone.0150881.s002.tif]

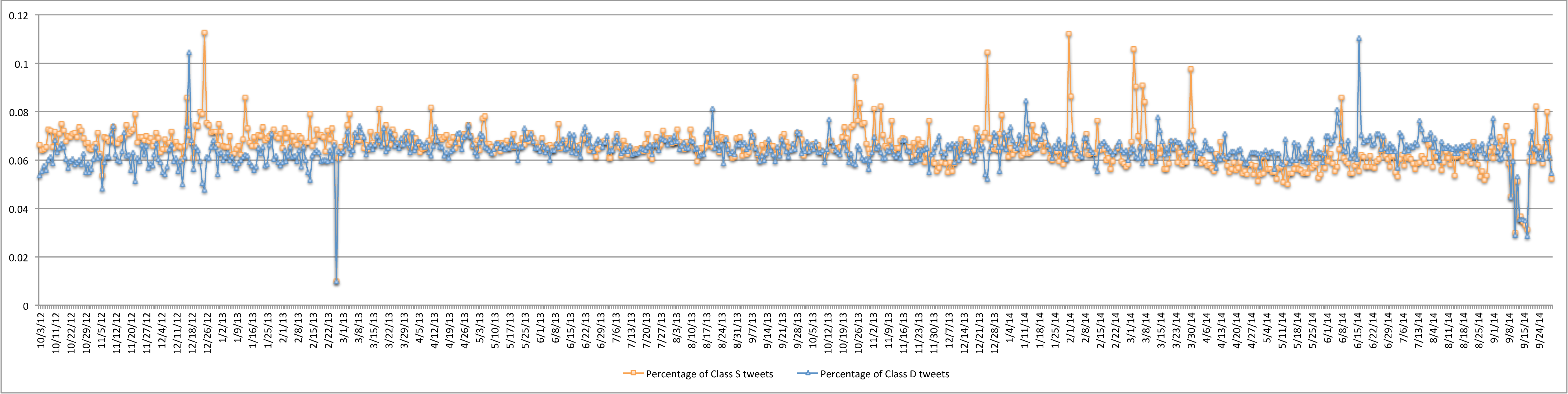

Supplement: S3 Fig — X axis: week. Y axis: Percentage computed as # Class S (or D) tweets in a day# First Person Tweets in the day×100%. (TIF) [file pone.0150881.s003.tif]

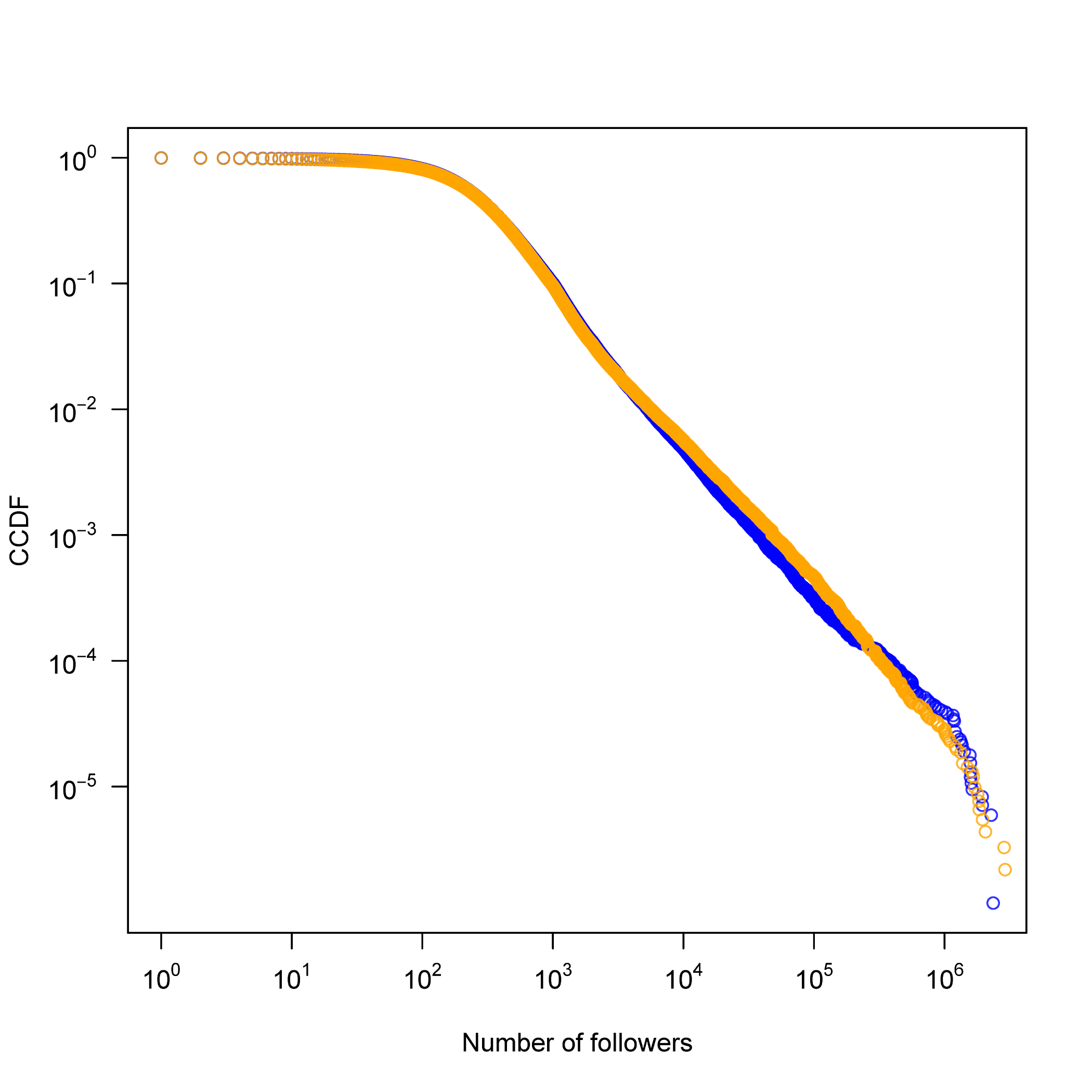

Supplement: S4 Fig — Yellow represents Class S users and blue represents Class D users. The two groups show unremarkable differences. (TIF) [file pone.0150881.s004.tif]

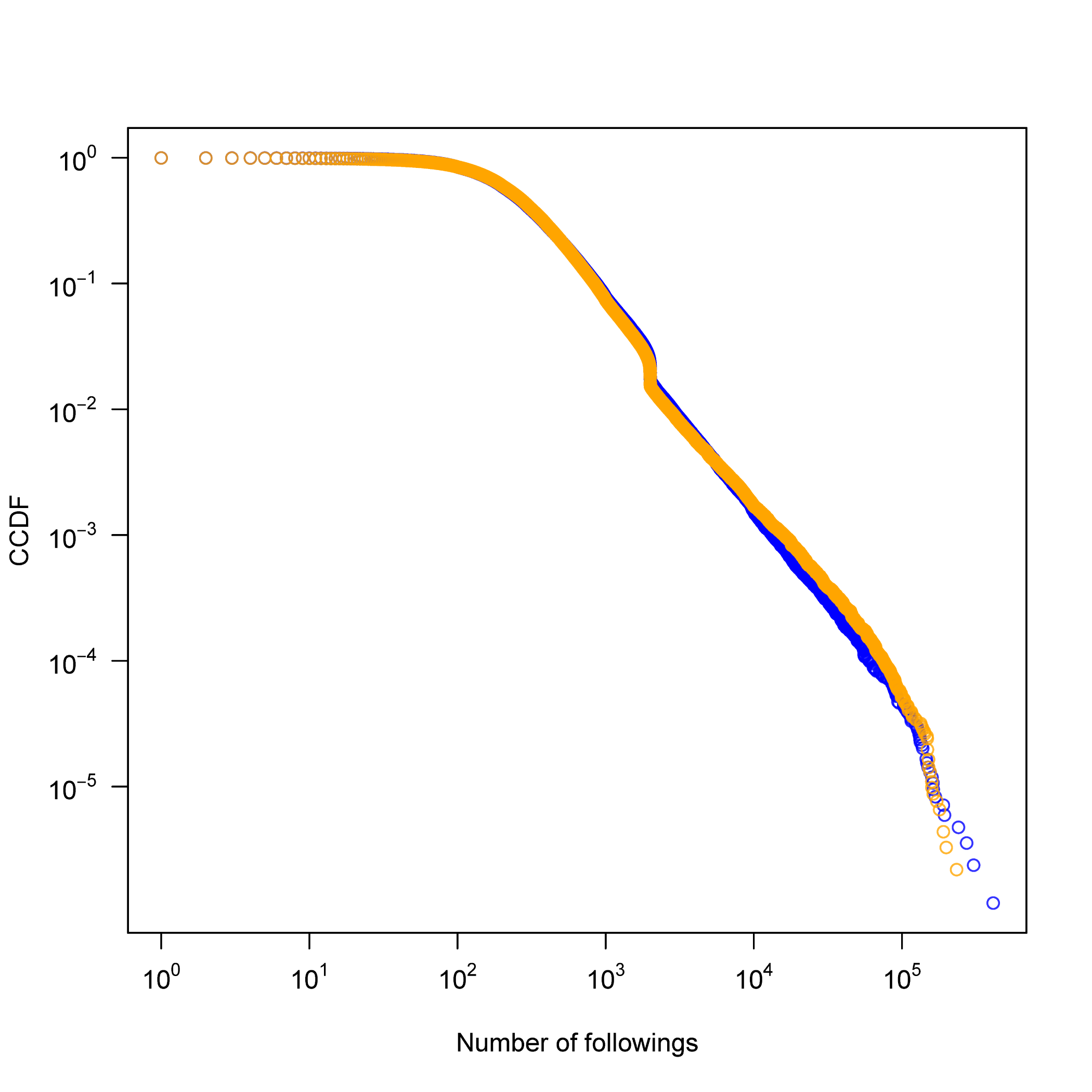

Supplement: S5 Fig — Yellow represents Class S users and blue represents Class D users. The two groups show unremarkable differences. (TIF) [file pone.0150881.s005.tif]
